# N-doped TiO_2_ nanocatalyst for one-pot synthesis of α,β-unsaturated esters *via* the Wittig reaction: a highly efficient and recyclable heterogeneous system

**DOI:** 10.1039/d5ra09170e

**Published:** 2026-02-26

**Authors:** Rohinee D. Hoval, Santosh T. Shinde, Anandrao A. Kale, Nanasaheb S. Gaikwad, Digambar B. Bankar, Nitin M. Thorat, Ramesh B. Gawade, Kaluram G. Kanade, Dinesh P. Amalnerkar

**Affiliations:** a Post Graduate Department and Research Centre of Chemistry, Annasaheb Awate College Manchar-410503 India drsantoshinde@gmail.com stshinde@aacmanchar.edu.in; b Pimpri Chinchwad University Pune-412106 India; c Post Graduate Department of Chemistry and Research Centre, R. B. Narayanrao Borawake College, Shrirampur (Autonomous) 413709 India; d Post Graduate Department of Chemistry, and Research Centre, Maharaja Jivajirao Shinde Mahavidyalaya Shrigonda Ahilyanagar-413701 India; e Rajmata Jijau Shikshan Prasarak Mandal's Arts, Commerce & Science College Landewadi, Bhosari Pune-411039 India

## Abstract

Wittig reactions represent one of the important tools for the stereoselective synthesis of substituted alkenes. In view of the importance of the Wittig reaction in organic synthesis, we have explored the Wittig reaction using a nitrogen-doped titanium dioxide (N-TiO_2_) nanocatalyst. The nitrogen doping of titanium dioxide was achieved *via* a sol–gel method employing triethylamine as the nitrogen source. The structural and morphological elucidation of the as-synthesized N-TiO_2_ nanocatalyst were carried out by X-ray diffraction (XRD), Scanning Electron Microscopy (SEM), Energy Dispersive X-ray Spectroscopy (EDX or EDS), X-ray Photoelectron Spectroscopy (XPS), Fourier-transform infrared spectroscopy (FTIR), high-resolution transmission electron microscopy (HRTEM) and Brunauer–Emmett–Teller (BET). The XRD analysis confirmed the formation of an anatase-dominated mixed phase of titanium dioxide, with an average crystallite size of approximately 10–20 nm, which is in good agreement with the particle size observed in the HRTEM analysis. The synthesized sample has a rough and porous surface morphology, with irregularly shaped particles showing substantial aggregation, according to the FESEM micrographs recorded. The average particle size of the observed surface characteristics is around 20 nm, suggesting that nanoscale texturing is appropriate for improved surface-related capabilities. A noticeable nitrogen signal in the elemental spectrum from EDX analysis demonstrated that nitrogen had been successfully incorporated into the TiO_2_ lattice. XPS analysis elucidated the efficient incorporation of nitrogen into the TiO_2_ lattice, involving the substitution of oxygen atoms, where nitrogen was identified to exist in both substitutional N(O–Ti–N) and interstitial N(Ti–O–N) forms. The as-synthesized N-TiO_2_ nanocrystalline material was employed as a heterogenous catalyst for the one-pot Wittig reaction involving aldehydes, α-halo esters, and triphenylphosphine to produce α,β-unsaturated esters in good yields by maintaining E-stereoselectivity at room temperature. The N-TiO_2_ nanocatalyst was recovered and recycled by a simple filtration technique. The structural confirmation of the synthesized Wittig product was done by ^1^H-NMR and ^13^C-NMR spectral techniques. The study establishes N-TiO_2_ as a robust and stable catalyst for the synthesis of α,β-unsaturated esters. The synthetic protocol presented herein offers several merits, including mild reaction conditions, high purity of synthesized products, facile catalyst separation, straightforward reaction workup and environmental benignity. These findings emphasize the potential of the nanostructured N-TiO_2_ material as a well-organized and sustainable heterogeneous nanocatalyst for the synthesis of valuable organic compounds.

## Abstract 2

This work reports an N-TiO_2_ nanoparticle-mediated Wittig reaction enabling stereoselective synthesis of (*Z*)-alkenes with high yield, simplified work-up, and excellent catalyst recyclability.

## Graphical abstract

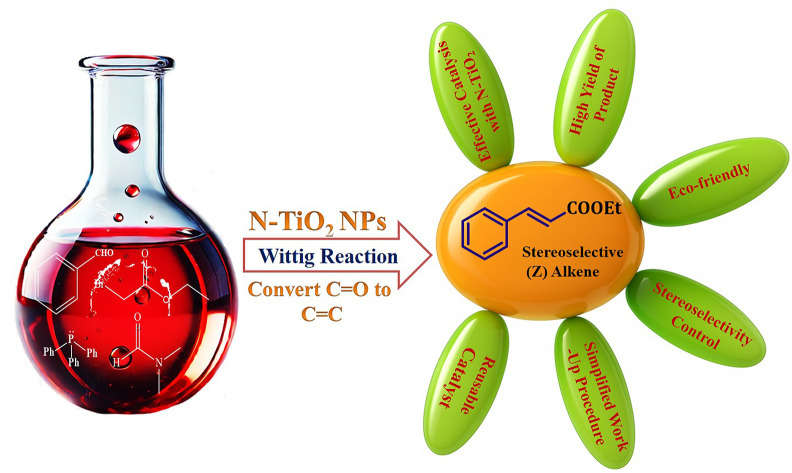

## 1. Introduction

Nowadays, nanocatalysis has gained significant attention in organic transformation due to a critical point of convergence between heterogeneous and homogeneous catalytic processes. In the chemical industry, catalysts are crucial because they contribute to more environmentally friendly and efficient operations. Catalysts are used in about 70–75% of industrial chemical processes in a variety of industries, including the production of fuels, polymers, medications, and agricultural chemicals.^1–6^ There are two primary types of catalysts: homogeneous catalysts, which are in the same state as the reacting substances, and heterogeneous catalysts, which are solid and act on their surface. Catalysts are crucial to science and technology because they help chemical reactions to produce more useful products, operate at lower temperatures, and control the formation of specific molecules in certain reactions. On the other hand, the benefits of heterogeneous catalysts include simple separation and recycling on reaction completion.^7–10^

Hence, owing to the significant role of solid materials as catalysts, researchers have increasingly focused on the use of transition metal oxides in heterogeneous organic transformations.^11^ For example, oxides such as ZnO, CuO/Cu_2_O, MnO_2,_ V_2_O_5_, Fe_2_O_3_/Fe_3_O_4_, Co_3_O_4_, TiO_2_, and CrO_3_ have been employed in various organic reactions. Among these, nanocrystalline titanium dioxide (TiO_2_) stands out due to its superior physicochemical properties, offering broader applications compared to other transition metal oxides. Nanocrystalline TiO_2_ is ideal for a variety of applications, such as organic synthesis, photocatalysis (environmental remediation), paints and coatings (pigments), sensors, photovoltaic cells, antimicrobial coatings, and cosmetics, due to its excellent stability and amphoteric nature.^12–16^ Doped TiO_2_ demonstrates that the phase composition, surface area, and shape all affect its photocatalytic activity. TiO_2_ with around 70% anatase and 30% rutile produced the greatest results and improved charge separation. The proper combination of phase and shape has a greater effect on performance, even though surface area is crucial. These characteristics make it possible to use it for organic transformation.^17–19^

One of the most challenging problems in organic chemistry is the formation of carbon–carbon double bonds in a certain way.^20–22^ In organic chemistry, the Wittig reaction is a powerful and efficient technique for creating carbon–carbon double bonds.^23,24^ It attracted a lot of interest from both synthetic and mechanistic perspectives. Several fluorinated chemicals and physiologically or biologically active molecules have been produced using the Wittig process to synthesise essential intermediates.^25–30^ In a traditional Wittig reaction, a phosphonium salt is prepared, a base is treated to produce an ylide, and then the phosphonium salt reacts with carbonyl compounds to produce an olefinic product. There are still some major disadvantages to these widely applicable techniques, such as the need for stepwise synthesis of ylide precursors under basic conditions.^31^

Over the past few decades, the formation of olefinic (C=C) bonds has attracted considerable attention, with the Wittig reaction being recognised as one of the most powerful and versatile methods for constructing such bonds. Recently, a Ph_3_As-catalyzed Wittig-type olefination reaction of aldehydes using diazoacetates has been described in which trisubstituted phosphine (Ar_3_P) was found to be an efficient organocatalyst for the synthesis of alkenes under mild, metal-free conditions. The reaction is readily performed in sodium dithionite (Na_2_S_2_O_4_) as the reagent.^32^ Other advances in phosphorus redox catalysis have demonstrated that both phosphine/phosphine oxide and phospholane/phospholane oxide systems can operate in a catalytic manner when combined with hydrosilane-based reductants such as phenylsilane. These systems enable the regeneration of active phosphine species from their oxidized forms, thereby sustaining a catalytic cycle that allows for effective olefination, including Wittig-type and related transformations, with significantly reduced phosphorus loading.^33–42^

Further, homogeneous catalysts such as Fe-phosphine,^43^ iron(ii) N-heterocyclic carbene complex,^44^ tributyl tellurium oxide dimer, dibutyl telluride^45^ have been used for the synthesis of olefins. Despite their high activity and selectivity, homogeneous catalysts have a number of drawbacks, including low thermal stability, susceptibility to oxygen and moisture, possible product contamination, and challenging recovery and recycling. In addition to raising operating costs, these disadvantages restrict their large-scale industrial uses by posing environmental and purification issues.

To overcome these challenges, researchers have been using heterogeneous catalysts more and more to get over such obstacles. The Wittig reaction's synthetic importance and broad use in creating carbon–carbon double bonds have also increased interest in creating effective heterogeneous catalytic systems designed especially for this transformation. Olefins were also synthesized using heterogeneous catalysts, including NAP-MgO,^46^ doped MgO,^47^ magnesium/lanthanum mixed oxide,^48^ silica-supported ruthenium nanoparticles,^49^ PE-supported arsine,^50^ and PEG-supported telluride.^51^ The reported works on the Wittig reaction are summarized in Table 1, which provides a comparative overview of various catalytic systems along with their reported yields and references.

**Table 1:** Comparative overview of reported catalytic systems for the Wittig reaction

Sr. no.	Catalytic system	Yield (%)	Ref. no.
1	Ph_3_As, Fe(TCP)Cl, toluene/H_2_O, 80 °C, Na_2_S_2_O_4_, N_2_, 8–12 h	71–99	32
2	Phosphine oxide, Et_3_N, PhSiH_2_, base, toluene, 100 °C, 24 h	41–95	33–36
3	Tributylphosphine oxide, PhSiH_3_, butylene oxide, dioxane, MWI, 150 °C, 1–3 h	30–88	39
4	Fe + phosphine, PPh_3_, iPr_2_Net, PhSiH_3_, toluene, 100 °C, 18 h	22–94	43
5	Iron(ii) N-heterocyclic carbene complex, ethyl diazoacetate, PPh_3_, solvent, RT, argon atm	>90	44
6	PEG-telluride, P(OPh)_3_, K_2_CO_3_, toluene	0–100	45 and 51
7	NAP-MgO, PPh_3_, DMF, RT	76–98	46
8	MgO, PPh_3_, DMF, RT	>90	47
9	Mg/La mixed oxide, PPh_3_, DMF, RT	49–99	48
10	Silica supported ruthenium nanoparticles, toluene, 80 °C, O_2_, 24 h	40–99	49
11	PE-supported arsine, Fe(TCP)Cl, PMHS, toluene, 110 °C, 12 h	0–100	50
12	P(i-PrNCH_2_CH_2_)_3_N, TMSEA, THF, RT, 24 h, 1 N HCl	79–90	52

However, some of these catalytic systems do have drawbacks such as the need for a large amount of catalyst, the usage of solvents and inert gases, longer reaction times, *etc.* Therefore, a straightforward and effective technique for the synthesis of α,β-unsaturated esters must be found.

In this particular context, adjustable electronic structure, chemical stability, and advantageous surface features of TiO_2_ and co-doped TiO_2_ have made them extremely promising heterogeneous catalysts. Due to their exceptional catalytic activity and selectivity, these materials have been used extensively in a variety of organic transformations. They are used in a wide range of reactions such as Suzuki–Miyaura coupling,^53^ Heck coupling,^54^ Mannich reaction,^55^ aldol condensation,^56^ Biginelli multicomponent reaction,^57^ Michael addition,^58^ and Knoevenagel condensation.^59^ Furthermore, they enhance the breakdown of organic dyes and enable efficient oxidation–reduction reactions through synergistic mechanisms due to their photocatalytic effectiveness under light irradiation. These characteristics demonstrate their promise as adaptable catalysts in green and sustainable chemical applications.^60,61^

Hence, owing to the wide catalytic applications of TiO_2_ materials, the present study reports the catalytic activity of nitrogen-doped TiO_2_ (N-TiO_2_) nanocatalysts in the Wittig reaction for the synthesis of α,β-unsaturated esters (olefins). The N-TiO_2_ nanocatalyst was synthesized and thoroughly characterized to confirm its structural and morphological features. The as-synthesized nanostructured N-TiO_2_ material was then employed as an heterogeneous catalyst in the Wittig reaction. The proposed methodology offers a simple and efficient heterogenous approach for olefination reactions, which could be potentially valuable for applications in the industrial point of view.

## 2. Experimental

### 2.1. Chemicals and materials

All the chemicals used, were purchased from Merck and SRL Chemicals and used without further purification. Loba Chemie silica gel (100–200 mesh) was used for column chromatography and thin-layer chromatography was performed on Merck-precoated silica gel 60-F254 plates. All the other solvents and chemicals were obtained from commercial sources and purified using standard methods.

### 2.2. Synthesis of pure TiO_2_ nanoparticles

Titanium dioxide (TiO_2_) nanoparticles were synthesized by well-known modified sol–gel method^62,63^ with slight modification. For this synthesis, 7.1 mL of titanium tetraisopropoxide (TTIP) was dissolved in ethanol and subsequently hydrolysed with distilled water under continuous magnetic stirring at room temperature for 4 h. After the reaction, the mixture was allowed to settle for 2 h, and the supernatant liquid was carefully decanted. The obtained solid mass was then cooled in an ice bath to maintain a temperature of 0–5 °C. Hydrogen peroxide was added to the cooled reaction mass, followed by stirring for 2 h to form a gel. The resulting gel was dried at 150 °C for 12 h and subsequently calcined at 500 °C to obtain crystalline TiO_2_ nanoparticles.

### 2.3. Synthesis of N doped TiO_2_ nanoparticles

N-TiO_2_ nanomaterials have been synthesized using a previously described technique with slight modifications.^64,65^ A series of nitrogen-doped TiO_2_ catalysts TN1, TN2, TN3, TN4, and TN5 were prepared with varying temperature 150 °C, 250 °C, 350 °C, 450 °C, 550 °C using a sol–gel method. Triethylamine was utilized as the nitrogen source, while titanium tetraisopropoxide (TTIP) served as the titanium source. In a typical synthesis, titanium tetraisopropoxide was dissolved in ethanol and then hydrolysed in distilled water under continuous stirring at room temperature for 4 h. The reaction mass was allowed to be settled for 2 h and the formed supernatant liquid was decanted carefully. Furthermore, to control the exothermic reaction, the reaction mixture was externally cooled using an ice bath before the addition of hydrogen peroxide solution along with the required amount of aqueous triethylamine solution, leading to the formation of an orange-yellow viscous gel. The formed nitrogen–titanium peroxide gel was subsequently dried in a hot air oven at 150 °C for 12 h. Finally, the dried powder material was calcined at 150 °C to 550 °C temperature under nitrogen atmosphere to yield the final nanocrystalline N-TiO_2_ material.

### 2.4. Synthesis of α,β-unsaturated esters *via* one-pot Wittig reaction catalysed by N doped TiO_2_ nanoparticles

For the synthesis of α,β-unsaturated esters *via* Wittig reaction, N-TiO_2_ nanocatalyst (1.2 mmol) was taken in 25 mL single neck round bottom flask containing reaction mixture of aldehyde (4.72 mmol), ethyl bromoacetate (4.72 mmol), and triphenylphosphine (4.72 mmol) in 5 mL of DMF. The mixture was stirred at room temperature under inert atmosphere until the reaction reached completion, as monitored by thin-layer chromatography (TLC). After completion of the reaction, the catalyst was separated by centrifugation and residual solid was washed with ethyl acetate. The water was then added to the filtrate, and the reaction mixture was extracted with ethyl acetate to isolate the organic phase. The combined organic layers were subsequently washed with water and brine, dried over anhydrous Na_2_SO_4_. To ensure the complete removal of DMF, the protocol involved an initial water addition followed by ethyl acetate extraction. The solvent was then evaporated under reduced pressure, and the resulting crude product was purified by column chromatography using silica gel (100–200 mesh) with an ethyl acetate/hexane gradient as the eluent to afford the pure product.^66^

### 2.5. Characterization techniques

X-ray diffraction (XRD) study of the powdered samples was done by using Rigaku Ultila IV (Cu K radiation, = 1.5406 Å). The morphological investigations were performed using Field Emission Scanning Electron Microscope (FESEM Model-Nova Nano SEM 450, Make-FEI). Loading of N on TiO_2_ nanoparticles was monitored using Energy Dispersive Spectrometer (EDS, Make: Bruker, Model: XFlash 6130). For confirmation of chemical composition, X-ray photoelectron spectroscopic (XPS) scans were recorded with XPS spectrophotometer (Thermo Fisher Scientific Instrument, UK) using monochromatic Al Kα with 6 mA beam current and 12 kV X-ray source. The morphological and structural features of powdered samples were analyzed using a Talos F200S G2 HRTEM at 200 kV. Nitrogen adsorption–desorption isotherms were measured using a NOVA Touch 1LX surface area and pore size analyzer (Quantachrome Instruments, USA). The as-synthesized organic products were spectrally characterized by Nuclear Magnetic Resonance (NMR) spectroscopy. ^1^H NMR spectra and ^13^C NMR spectra were recorded using CDCl_3_ and DMSO-d_6_ solvents on Ascend 500 MHz Bruker NMR spectrometer. FT-IR spectra were recorded on the IR Prestige-21 SHIMADZU instrument using a KBr disc or pellet and are reported in terms of frequency of absorption (cm^−1^). Melting points (MP) are uncorrected and were recorded on a Thomas-Hoover Unimelt capillary melting point.

## 3. Results and discussion

### 3.1. Crystallographic analysis by XRD

**Fig. 1:**
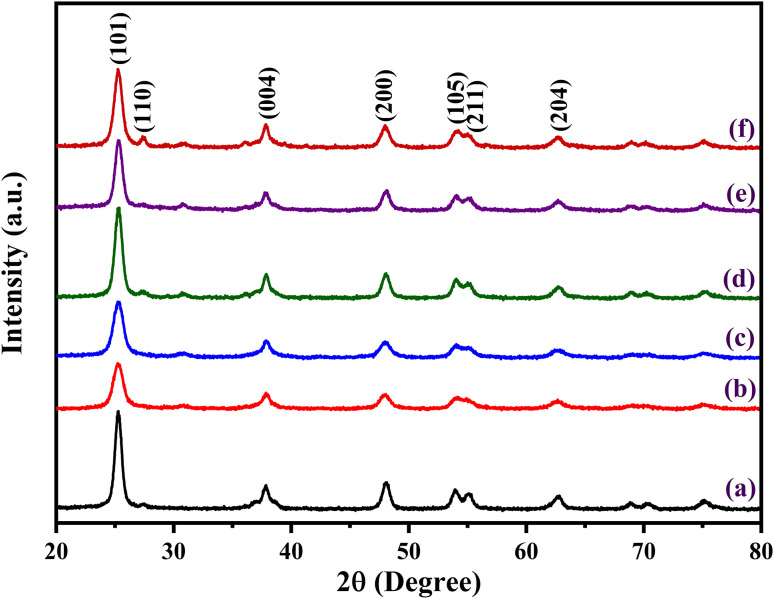
X-ray diffraction patterns of (a) TN0 (TiO_2_), (b) TN1 (150), (c) TN2 (250) (d) TN3 (350) (e) TN4 (450) (f) TN5 (550).

Different phase features are highlighted in the XRD pattern, which compares undoped and N-doped TiO_2_ samples. Using a powder X-ray diffraction pattern, the phase purity and phase formation of synthesised materials were examined (Fig. 1). With a prominent diffraction peak seen at 2*θ* ≈ 25.28°, which corresponds to the (101) plane of anatase TiO_2_ (JCPDS card no. 21-1272), all six samples primarily display the anatase phase. Additional reflections that further support the existence of additional anatase planes are (004), (200), (105), (204), and (116). A modest rutile component is indicated by a faint diffraction peak at 2*θ* ≈ 27.42° that is indexed to the (110) plane of the rutile phase (JCPDS card no. 21-1276). With an estimated composition of anatase and rutile phases, the material is thus classified as predominantly anatase with a tiny percentage of rutile. The Scherrer formula is used to determine the crystallite size from each (101) peak in the XRD pattern.^67^ The average crystalline size of undoped TiO_2_, N-TiO_2_ (150), N-TiO_2_ (250), N-TiO_2_ (350), N-TiO_2_ (450), and N-TiO_2_ (550) are 11.47, 7.85, 7.58, 10.45, 10.10, and 9.46 nm, respectively. Significant alterations in the XRD pattern are noted with nitrogen doping. A decrease in crystallite size and an increase in surface area is suggested by the diffraction peaks being wider and less intense. There are also noticeable little peak changes, which are explained by the lattice distortion brought on by the addition of N. According to XRD analysis, N-doped TiO_2_ provides better structural characteristics that are perfect for improved performance in organic processes, especially when exposed to visible light.

### 3.2. Nanostructural surface investigation by FESEM

**Fig. 2:**
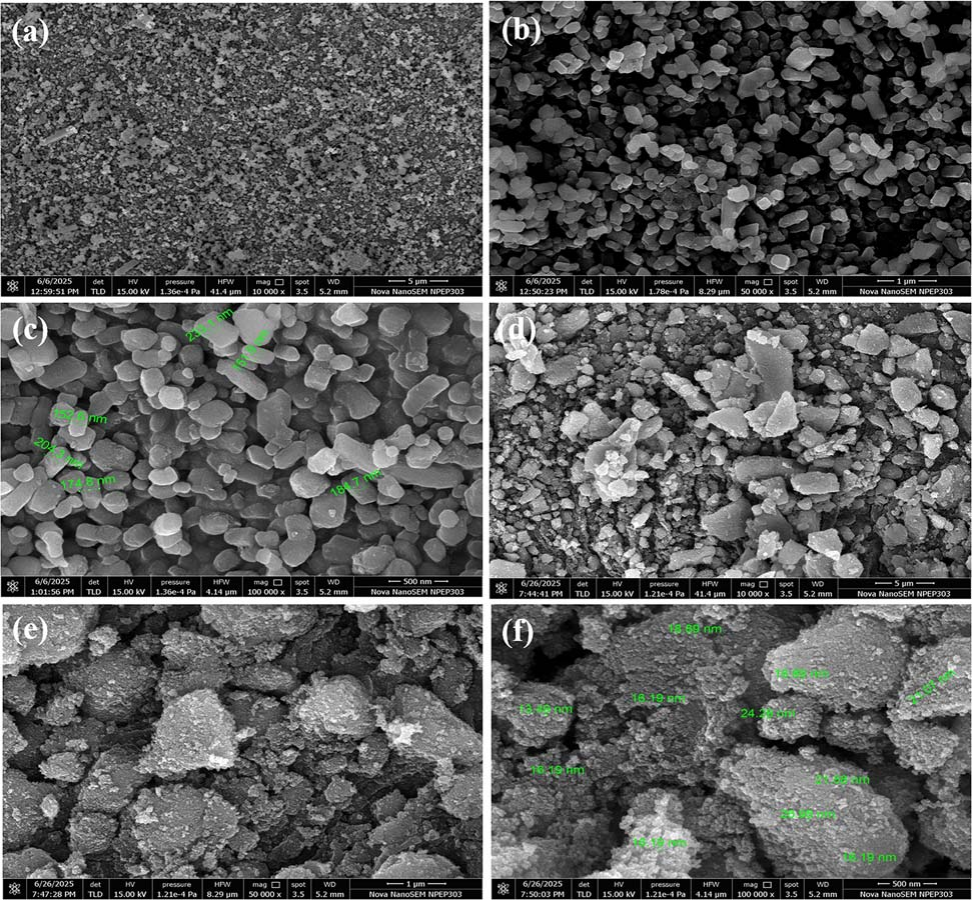
FESEM images: (a–c) TiO_2_ and (d–f) N-TiO_2_.

The FESEM images displayed in Fig. 2 show notable morphological changes when TiO_2_ is doped with nitrogen. The uniformly dispersed, well-defined, smooth-surfaced spherical to rod-like particles in the pure TiO_2_ sample show a high degree of crystallinity and little aggregation. This uniform shape is characteristic of undoped TiO_2_ that has been produced under controlled circumstances, which permits effective packing but has a comparatively smaller surface area. On the other hand, the N-doped TiO_2_ sample exhibits rough, porous surfaces and agglomerated, irregularly shaped particles. This alteration is explained by the addition of nitrogen to the TiO_2_ lattice during synthesis, which causes lattice strain, interferes with crystal growth, and encourages the development of defect-rich structures. The material's surface area is increased by a higher degree of aggregation and porosity, which can greatly boost its photocatalytic activity when exposed to visible light. When nitrogen increases surface roughness, it not only lowers the bandgap energy of TiO_2_ but also enhances charge separation and active site availability. For cutting-edge uses including pollutant degradation, water splitting, and solar-driven processes, N-TiO_2_'s modified morphology is therefore essential. Its superior performance over pure TiO_2_ is directly attributed to this morphological difference.

### 3.3. Elemental analysis by EDS

**Fig. 3:**
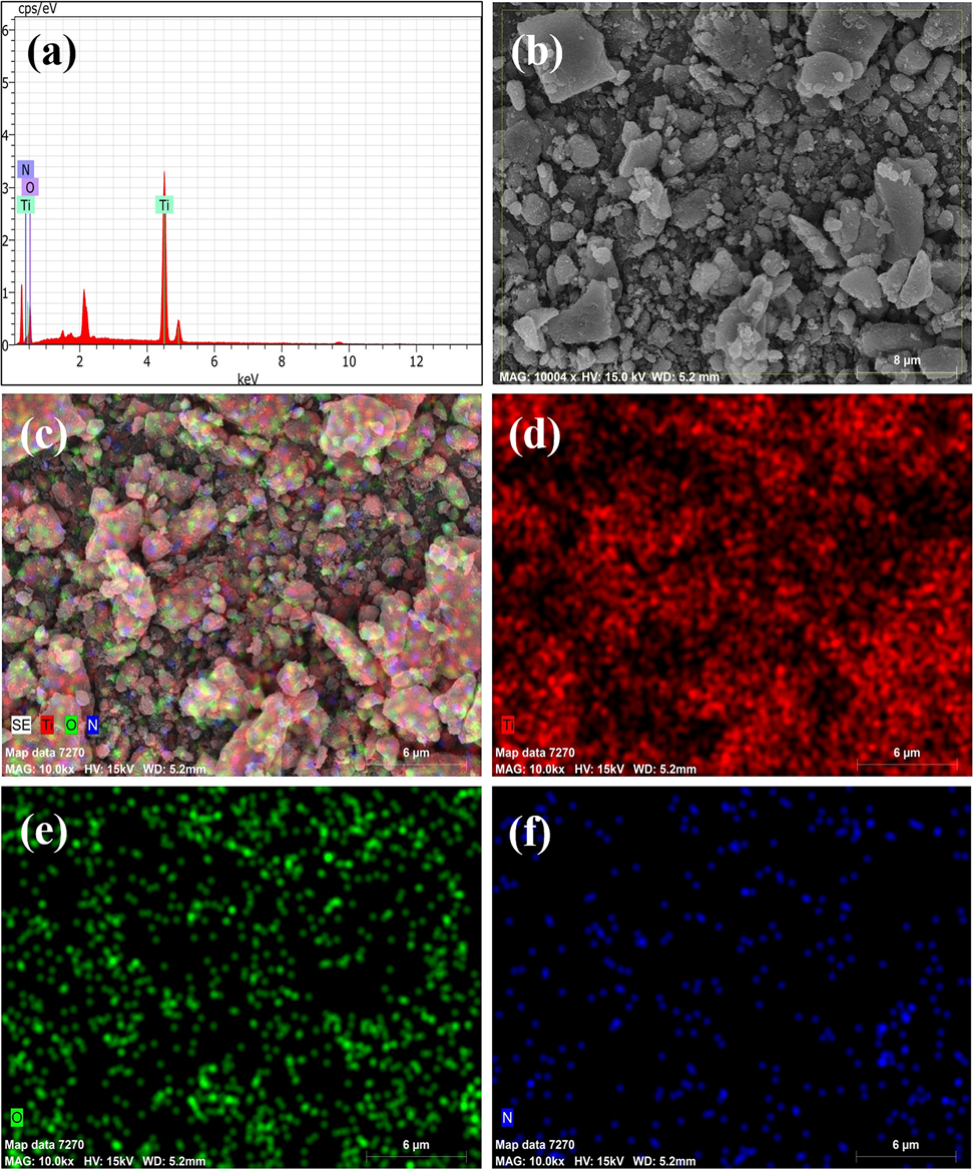
(a) EDS spectrum, (b) EDS element layered image, (c) EDS element mapping images, (d) Ti (e) O (f) N of N-doped TiO_2_ (TN3 350) nanoparticles.

The SEM morphology and elemental analysis of nitrogen-doped TiO_2_ nanoparticles are displayed in Fig. 3. Agglomerated, rough-surfaced grains characteristic of doped TiO_2_ are visible in the SEM picture (b), suggesting that nitrogen inclusion has interrupted crystal formation. The existence of Ti, O, and N is confirmed by the EDX spectrum (a), which shows atomic percentages of 43.68%, 56.07%, and 0.39 percent, respectively. The clear peak and mapping confirm successful N-doping despite the low nitrogen concentration. The distribution of titanium, oxygen, and nitrogen is depicted in elemental maps (d–f). While nitrogen is found sparingly and unevenly, titanium and oxygen are consistently distributed. By enhancing surface flaws and active sites, this elemental incorporation and surface morphological improvement enable enhanced photocatalytic capabilities under visible light.

### 3.4. Elemental and chemical state evaluation (XPS)

**Fig. 4:**
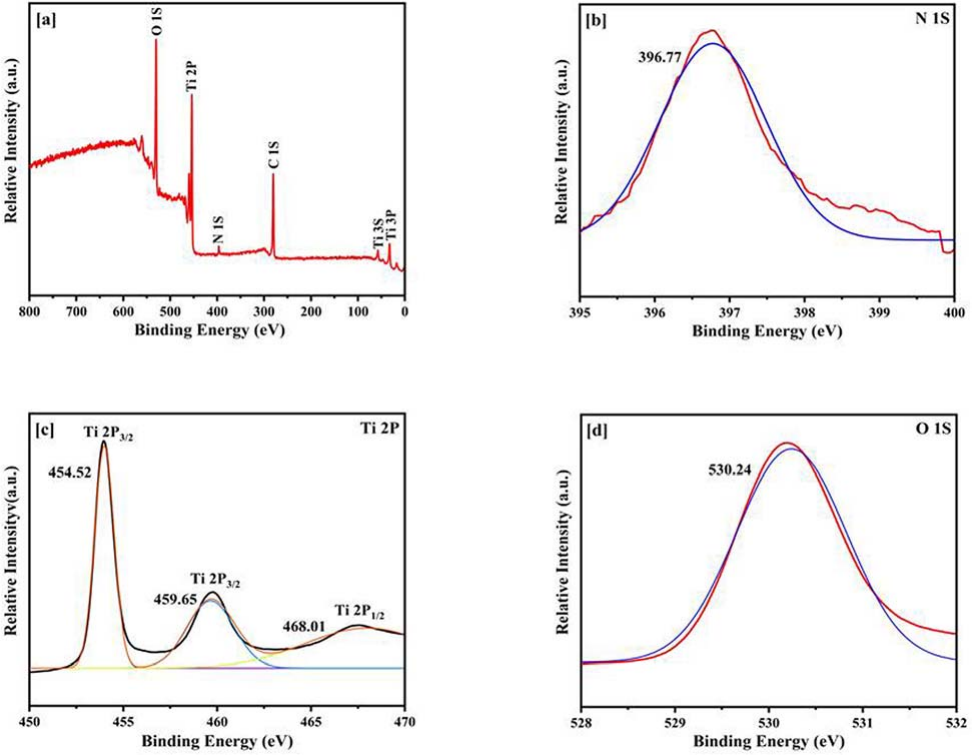
XPS analysis of N-TiO_2_ spectra (a) survey scan (b) high resolution spectrum of N 1s region (c) high resolution spectrum of Ti 2p region (d) high resolution spectrum of O 1s region.

The presence of Ti, O, N and C in the survey scan demonstrated that nitrogen had been successfully incorporated into the TiO_2_ lattice, as validated by X-ray Photoelectron Spectroscopy (XPS) examination of N-doped TiO_2_ (Fig. 4). Surface contamination from adventitious carbon, which might come from the XPS instrument or exposure to ambient air, is most likely the cause of the detected carbon peak in the XPS spectra.^68^ A noticeable peak at around 396.5 eV in the high-resolution N 1s spectrum was a sign of substitutionally doped nitrogen (N–Ti–O), which adds mid-gap states above the valence band to improve absorption of visible light. The existence of interstitial nitrogen (Ti–O–N) or adsorbed NO_*x*_ molecules is suggested by minor peaks at around 399–401 eV. Oxygen vacancies produced during doping were indicated by the main peaks in the Ti 2p region at 458.5 eV (Ti^4+^ 2p_3/2_) and 464.2 eV (Ti^4+^ 2p_1/2_), as well as a shoulder at ∼457.3 eV due to Ti^3+^ states. These Ti^3+^ species increase catalytic efficiency and make charge separation easier. The O 1s spectrum showed a secondary component at ∼532.8 eV caused by surface hydroxyl groups or adsorbed water, which are known to improve surface reactivity and hydrophilicity, and a significant signal at ∼530.5 eV corresponding to lattice oxygen (O^2−^ in Ti–O). When combined, these spectrum characteristics confirm the existence of nitrogen doping, defect formation, and surface functionalization all of which work in synergy to improve photocatalytic activity in visible light.^69^

### 3.5. Functional group analysis by FT-IR

**Fig. 5:**
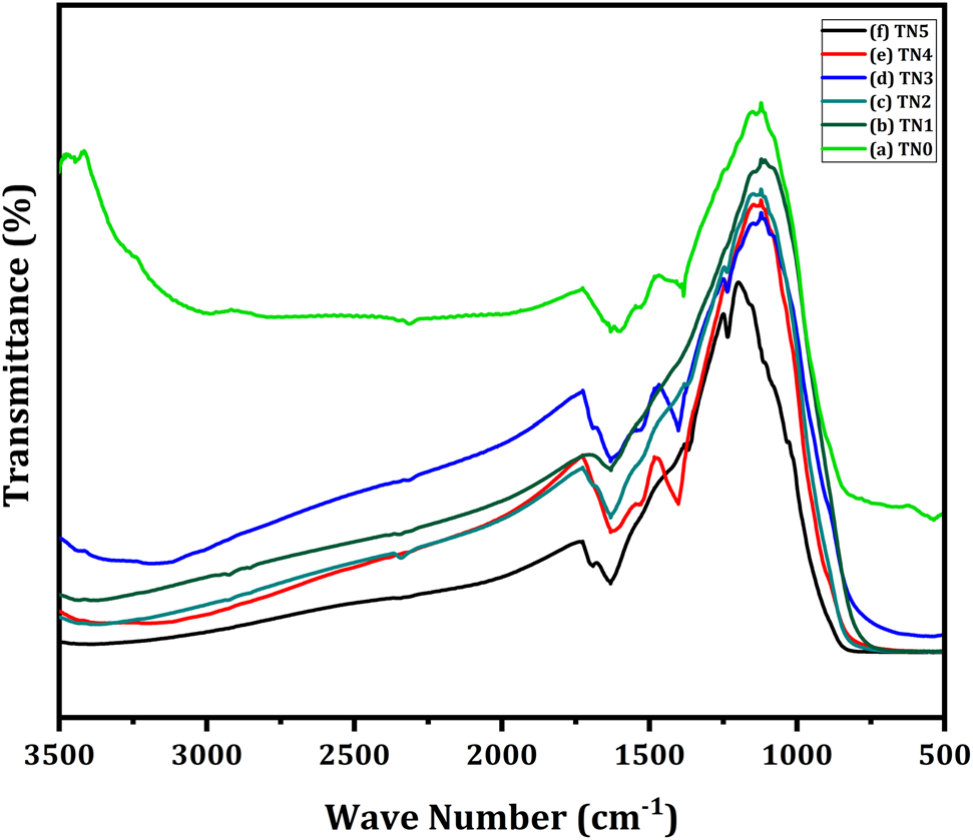
FTIR spectra of (a) TN0 (TiO_2_), (b) TN1 (150), (c) TN2 (250), (d) TN3 (350) (e) TN4 (450) (f) TN5 (550).

Numerous vibrational attributes that indicate successful TiO_2_ framework building and possible nitrogen incorporation are visible in the FTIR spectra of undoped and nitrogen-doped TiO_2_ samples (Fig. 5). The development of the TiO_2_ lattice structure is confirmed by a noticeable and wide absorption band seen in the 700–900 cm^−1^ range, which is associated with Ti–O–Ti and Ti–O stretching vibrations. A peak at about 1630 cm^−1^ is thought to be caused by the bending vibration of H–O–H bonds, which are produced by surface-adsorbed water molecules. Notably, significant absorption bands in the 1100–1300 cm^−1^ region are seen in samples TN1, TN2, TN3, TN4, TN5 which is due to successful incorporation of nitrogen in TiO_2_ matrix.^70^ Altogether, the spectrum characteristics validate the structural soundness of TiO_2_ as well as the successful alteration of its chemical environment by nitrogen doping.

### 3.6. Crystallographic and lattice structure analysis by HRTEM

**Fig. 6:**
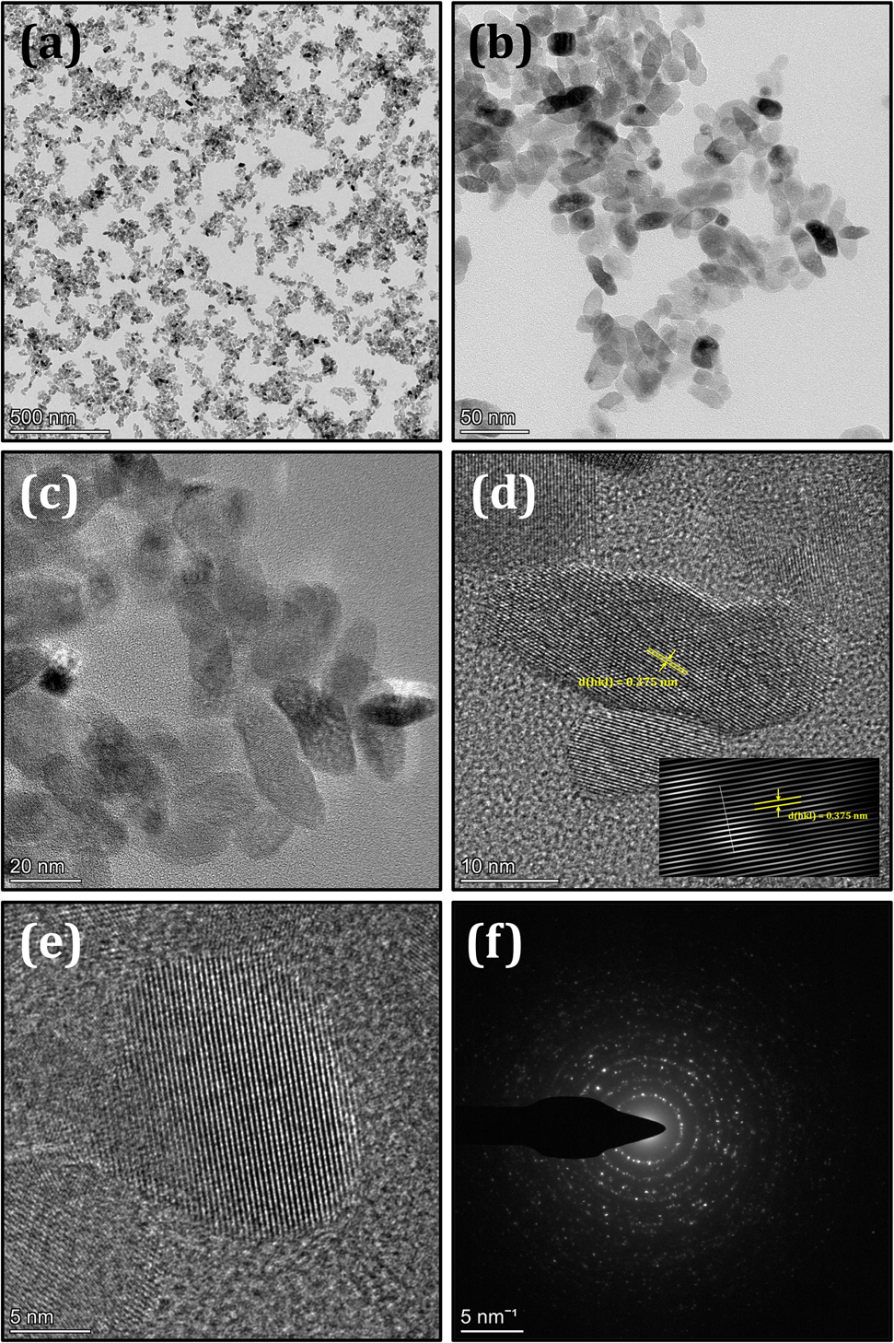
(a–c) TEM images of N-doped TiO_2_ nanoparticles showing their morphology, (d and e) HRTEM images showing clear lattice fringes (∼0.37 nm) and (f) SAED pattern confirming the crystalline nature.

The morphological and structural features of the synthesized N-TiO_2_ were systematically examined using HRTEM, as shown in Fig. 6(a–f). The low-magnification TEM image reveals densely distributed nanoparticles forming agglomerated clusters, which is typical for nanoscale TiO_2_ due to high surface energy. Higher magnification images indicate irregular to slightly elongated particles with sizes 10–20 nm, confirming the successful formation of nanostructured material after nitrogen incorporation. The HRTEM analysis displays well-resolved lattice fringes with an interplanar spacing of approximately 0.375 nm, suggesting high crystallinity and preservation of the TiO_2_ crystal lattice. The ordered atomic arrangement further indicates uniform crystal growth without significant structural distortion after doping. Moreover, the SAED pattern exhibits clear concentric diffraction rings with bright spots, confirming the polycrystalline nature of the synthesized N-doped TiO_2_. These observations collectively demonstrate that nitrogen doping maintains structural integrity while producing highly crystalline nanoscale TiO_2_, which is advantageous for enhanced catalytic and optoelectronic performance.

### 3.7. Surface area and porosity analysis by BET

**Fig. 7:**
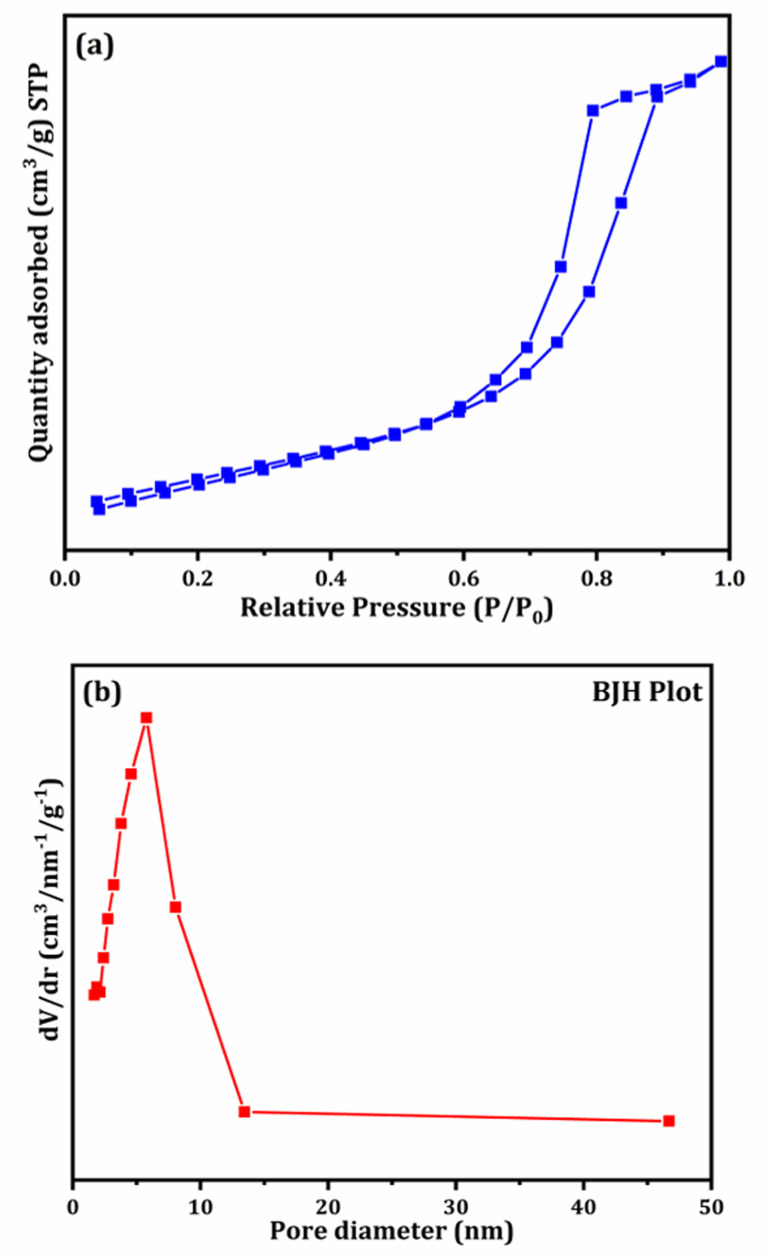
(a) Nitrogen adsorption–desorption isotherm and (b) BJH pore size distribution plot of N-TiO_2_ (TN3) nanoparticles.

The surface characteristics and pore structure of the synthesized N-TiO_2_ were examined using N_2_ adsorption–desorption measurements at 77 K. The adsorption isotherm Fig. 7(a) corresponds to a Type IV isotherm as per IUPAC classification, which is typical of mesoporous materials. A distinct H_3_/H_4_ hysteresis loop observed in the relative pressure (*P*/*P*_0_) range of 0.6–0.9 further confirms the presence of a well-developed mesoporous framework. The specific surface area calculated using the multi-point BET method was found to be 71.8654 m^2^ g^−1^, indicating substantial surface availability. The total pore volume for pores below 76.12 nm was determined to be 0.2011 cm^3^ g^−1^ at *P*/*P*_0_ = 0.987. BJH analysis revealed an average pore radius of 5.597 nm, corresponding to an average pore diameter of approximately 11.19 nm, which clearly lies within the mesoporous range. The BJH pore size distribution curve Fig. 7(b) exhibits a prominent peak in the 5–12 nm range, consistent with the calculated average pore diameter and indicating a relatively uniform pore structure. The combination of appreciable surface area and well-defined mesoporosity is advantageous for catalytic applications, as it enhances the availability of active sites and facilitates efficient diffusion of reactant molecules and reaction intermediates.

### 3.8. Catalytic activity of N-TiO_2_ nanomaterial for Wittig reactions

**Scheme 1:**
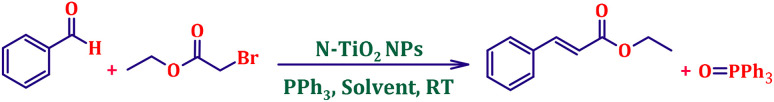
Model one-pot Wittig reaction using N-TiO_2_ nanocatalyst.

Following the characterization of the catalyst, its catalytic activity was evaluated under optimized experimental conditions. A model reaction was carried out between benzaldehyde and ethyl bromoacetate in the presence of triphenylphosphine, using N-doped TiO_2_ as the catalyst to assess its efficiency (Scheme 1).

**Scheme 2:**
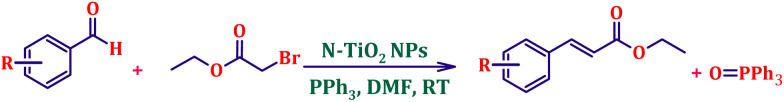
One-pot synthesis of α,β-unsaturated esters *via* Wittig olefination of aromatic aldehydes with ethyl bromoacetate using N-doped TiO_2_ nanoparticles.

The model reaction was initially performed without a catalyst, and under these conventional conditions, no product formation was observed (Table 2, entry 1). This clearly indicates that the presence of a catalyst is essential for the reaction to proceed. To evaluate the catalytic efficiency, a series of catalysts were tested under identical conditions, as summarized in Table 2 (entries 2–8). Among the examined catalysts, N-doped TiO_2_ nanoparticles (TN3 (350)) exhibited the highest catalytic activity, yielding 89% of the desired product within 14 h. In contrast, undoped TiO_2_ (TN0) and commercial TiO_2_ displayed significantly lower efficiencies, confirming the beneficial role of nitrogen doping and optimized calcination temperature in enhancing catalytic performance.

**Table 2:** Comparative study of catalysts efficiency for the stereoselective synthesis of α,β-unsaturated esters in DMF solvent^a^

Entry	Catalyst	Time (h)	Yield^b^ (%)
1	—	48	—
2	Commercial TiO_2_	38	11
3	TN0 (TiO_2_)	32	15
4	TN1 (150)	23	60
5	TN2 (250)	25	72
6	TN3 (350)	14	89
7	TN4 (450)	16	85
8	TN5 (550)	18	79

a Reaction conditions: benzaldehyde (4.72 mmol), ethyl bromoacetate (4.72 mol), PPh_3_ (4.72 mol), catalyst (1 mmol), DMF (5 mL), RT.

b Isolated yield.

The superior catalytic activity of the N-doped TiO_2_ (TN3-350) nanocrystalline material can be attributed to its well-defined structural and surface characteristics. XRD results reveal a crystallite size of 10–20 nm, which is consistent with FESEM and TEM observations, confirming the formation of finely dispersed nanocrystals. Such nanoscale dimensions markedly increase the surface-to-volume ratio, ensuring a higher availability of active surface sites for catalytic interaction. BET analysis further demonstrates enhanced surface area and porosity, which improve reactant adsorption and facilitate smoother diffusion of the phosphonium ylide and carbonyl substrates across the catalyst surface. These textural advantages promote efficient contact between reacting species, thereby supporting the olefination pathway. Additionally, TiO_2_ inherently exhibits amphoteric behavior, providing both acidic and basic sites that aid in substrate activation. Nitrogen incorporation modifies the surface electronic environment, generating electron-rich centers and defect sites that enhance the basic character of the material. This increase in surface basicity assists in stabilizing reactive intermediates and promotes the nucleophilic addition step of the Wittig reaction, collectively establishing N-doped TiO_2_ as a rationally designed and effective catalytic system.

Inspired by the first results, a deeper investigation was conducted, and the results showed that N-doped TiO_2_ had a substantially greater catalytic performance than undoped TiO_2_ by conducting the model reaction at varying catalyst concentrations of 5, 10, 15, 20, 25, and 30 mol% of TN3 (350). As summarised in Table 3, the corresponding product yields were 59, 72, 87, 89, 92, and 92%, respectively. The yield increased steadily with increasing catalyst concentration up to 25 mol%, beyond which no significant improvement was observed. Therefore, 25 mol% of TN3 (350) was considered the optimal catalyst loading for this reaction (Table 3, entry 4).

**Table 3:** Screening of TN3 catalyst concentrations for stereoselective α,β-unsaturated ester formation^a^

Entry	Amount of catalyst (mol%)	Yield^b^ (%)
1	5	59
2	10	72
3	15	87
4	20	89
5	25	92
6	30	92

a Reaction conditions: benzaldehyde (4.72 mmol), ethyl bromoacetate (4.72 mmol), PPh_3_ (4.72 mmol), DMF (5 mL), RT, 16 h.

b Isolated yield.

To check the influence of solvent on efficiency of model reaction in 25 mol% TN3 (350) catalyst, a variety of solvents, including toluene, tetrahydrofuran, 1,4 dioxane, acetonitrile, DMF, ethanol, methanol, dichloromethane, and DMSO, were utilised (Table 4, entries 1–9). However, it was evident that the solvent had a significant impact on these reactions, and DMF produced higher yields of the intended product with superior stereoselectivity. Under the identical reaction conditions, tetrahydrofuran, acetonitrile and DMSO also produced good yields, but they performed marginally worse than DMF.

**Table 4:** Screening of solvents for the synthesis of α,β-unsaturated esters^a^

Entry	Solvent	Yield^b^ (%)
1	Toluene	25
2	Tetrahydrofuran	79
3	1,4 Dioxane	68
4	Acetonitrile	75
5	DMF	92
6	Ethanol	32
7	Methanol	29
8	Dichloromethane	48
9	DMSO	87

a Reaction conditions: benzaldehyde (4.72 mmol), ethyl bromoacetate (4.72 mol), PPh_3_ (4.72 mol), catalyst TN3 (350) (25 mol%), solvent (5 mL), RT, 16 h.

b Isolated yield.

The reaction was then carried out at different temperatures, from room temperature to 100 °C, in order to examine the impact of reaction temperature (Table 5, entries 1–6). The reaction conducted at room temperature had the highest yield of all of these, suggesting that it was the most advantageous temperature in comparison to the others.

**Table 5:** Effect of temperature on the synthesis of α,β-unsaturated esters^a^

Entry	Temperature (°C)	Yield^b^ (%)
1	−5 to 0	27
2	RT	92
3	40	92
4	60	94
5	80	94
6	100	92

a Reaction conditions: benzaldehyde (4.72 mmol), ethyl bromoacetate (4.72 mol), PPh_3_ (4.72 mol), catalyst TN3 (350) (25 mol%), solvent (5 mL), RT, 16 h.

b Isolated yield.

**Fig. 8:**
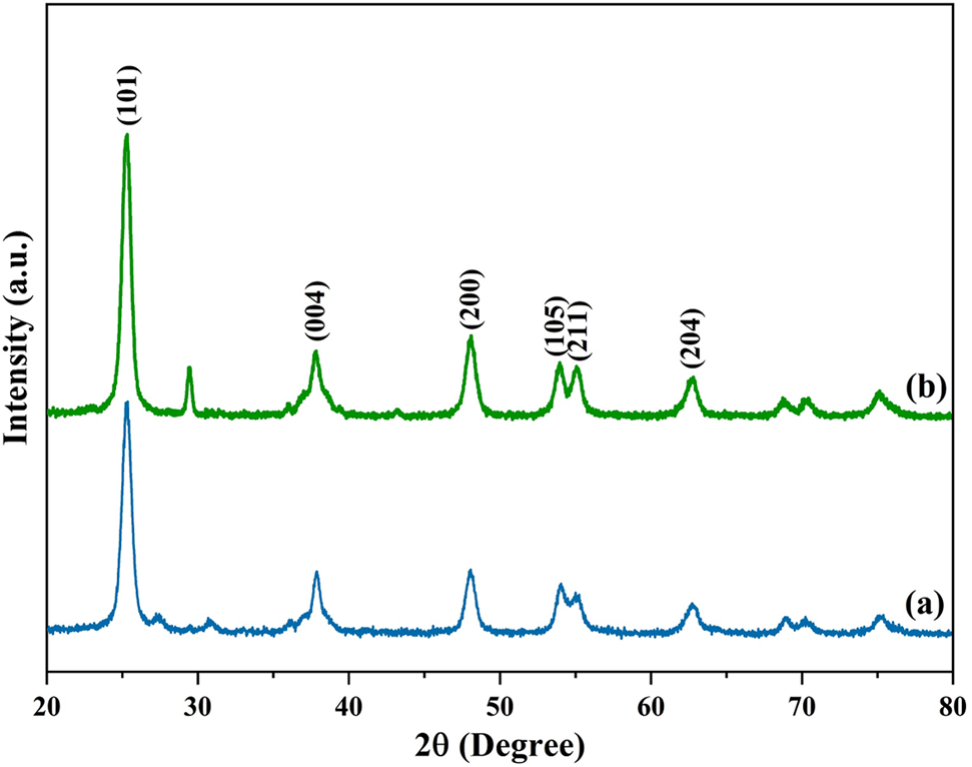
XRD of recycled N-TiO_2_ catalyst after 5 cycles.

**Fig. 9:**
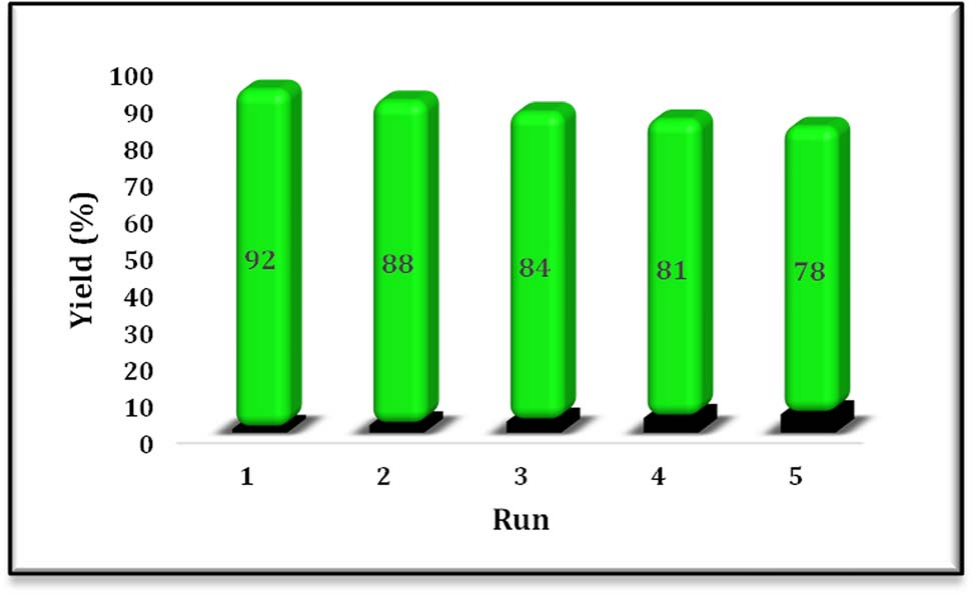
Reusability of catalyst TN3 (350); reaction condition: benzaldehyde (4.72 mmol), ethyl bromoacetate (4.72 mmol), PPh_3_ (4.72 mmol), catalyst TN3 (350) (25 mol%), DMF (5 mL).

After establishing the optimized reaction conditions for the synthesis of α,β-unsaturated esters, the reusability of the N-doped TiO_2_ catalyst was evaluated. The Wittig reaction was carried out under the optimized conditions for five consecutive cycles to assess the catalyst's stability and recyclability. Following each cycle, the catalyst was filtered out, cleaned of organic residues using ethanol and water. The catalyst was dried and calcined at 350 °C and used again. The yield of the intended alkene product in each cycle was used to evaluate the catalytic performance. Fig. 9 illustrates how the Wittig product's yield progressively dropped from 92% in the first cycle to 78% in the fifth. This decrease in efficiency may be caused by organic byproducts adhering to active sites, partial catalyst deactivation, or minor structural alterations brought on by frequent usage. Fig. 8 shows the catalyst's structural integrity was verified throughout recycling by XRD analysis conducted both before and after reaction. Indicating that the catalyst maintains its structural framework even after repeated usage, the comparison of the XRD patterns reveals that the catalyst's crystallinity and phase structure stayed mostly unaltered. Surface fouling or decreased active surface area, however, might be the cause of mild peak widening or intensity reduction. Overall, the catalyst exhibits reusability, maintaining significant activity up to five cycles.

To further explore the scope and versatility of the optimized reaction conditions, the Wittig reaction was extended to a range of substituted benzaldehydes using triphenylphosphine, ethyl bromoacetate, and DMF as the solvent at room temperature to produce a series of novel olefins and successfully extending the applicability of the optimised reaction conditions (TN3 (350)) leading good to excellent yields as shown in Table 6. Table 6 provides an overview of the corresponding results from the extensive characterisation of the synthesised compounds using a variety of spectroscopic techniques. The SI contains detailed spectroscopic information about the as-synthesised organic materials substances.

**Table 6:** Synthesis of α,β-unsaturated esters *via* the Wittig reaction under optimized reaction conditions using various substituted benzaldehydes^a^ (Scheme 2)

Entry	Aldehyde	Product	Yield^b^ (%)
1	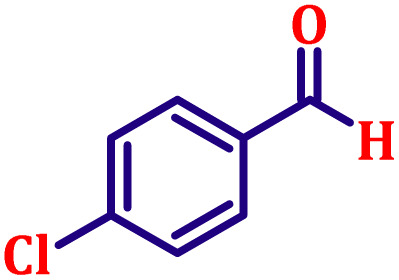	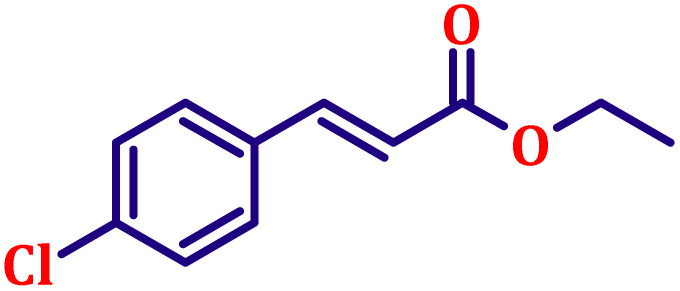	80
2	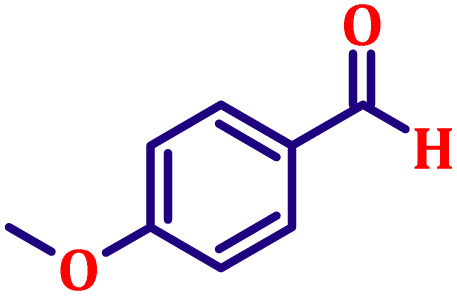	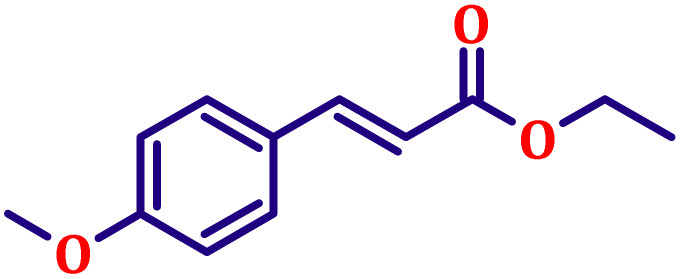	82
3	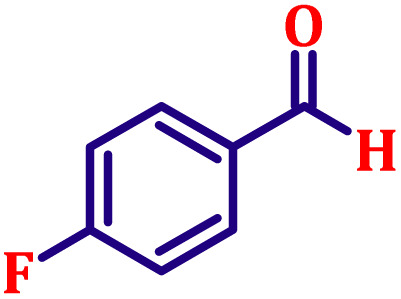	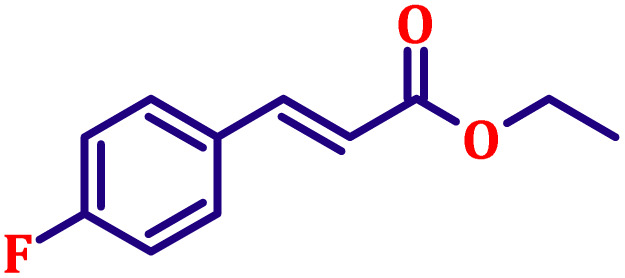	86
4	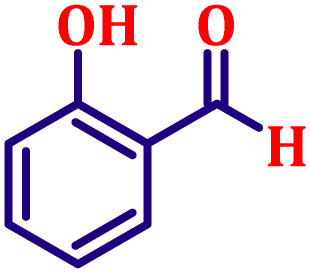	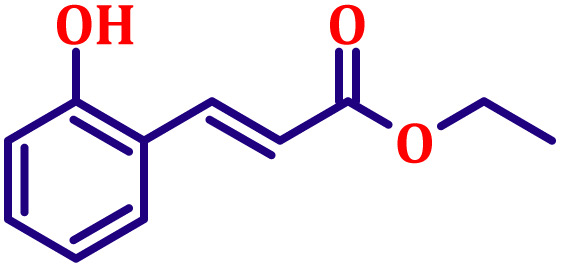	80
5	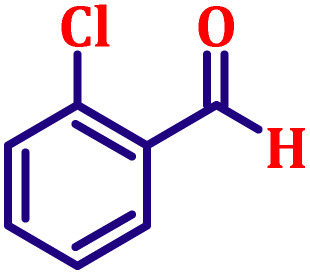	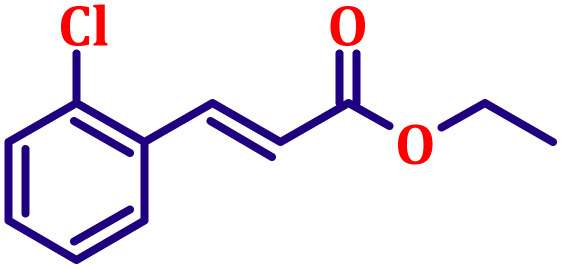	84
6	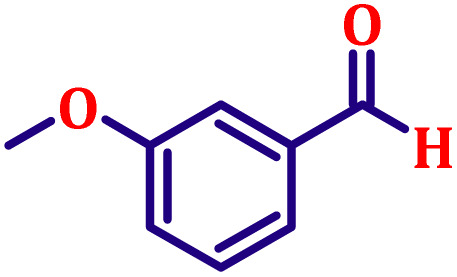	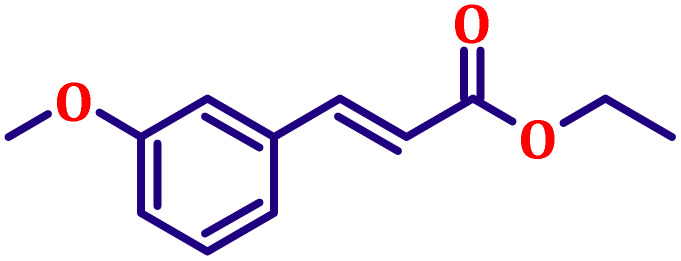	80
7	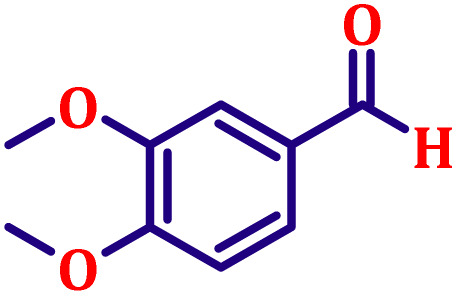	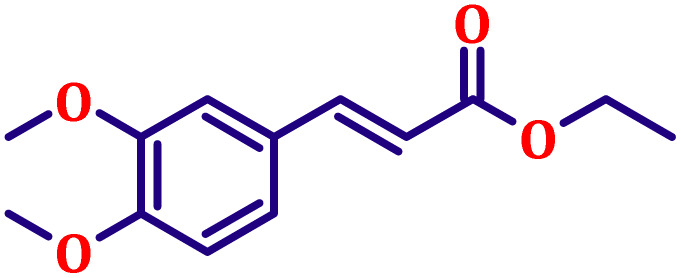	84
8	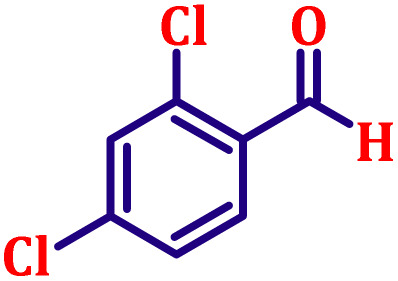	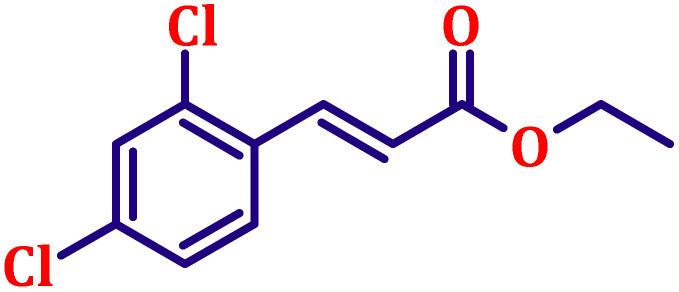	72
9	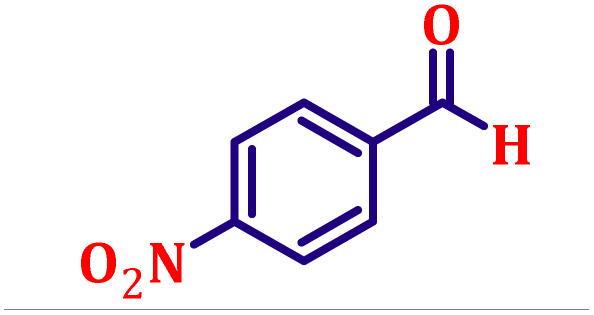	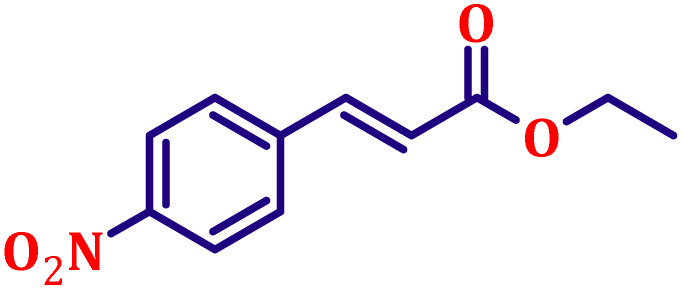	88
10	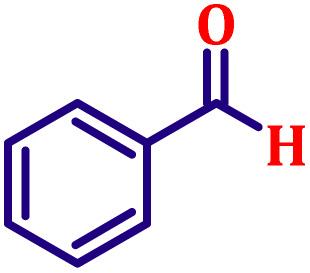	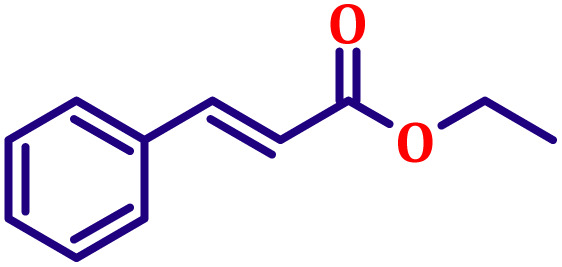	92
11	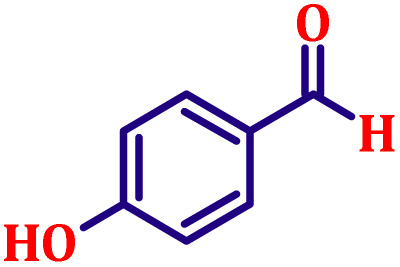	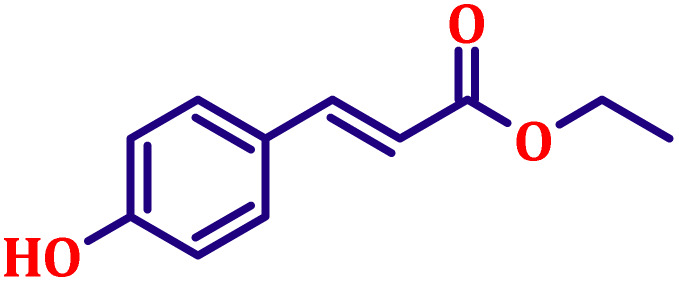	75
12	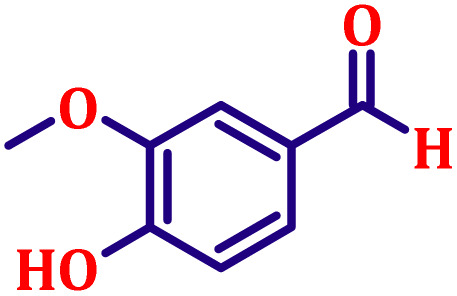	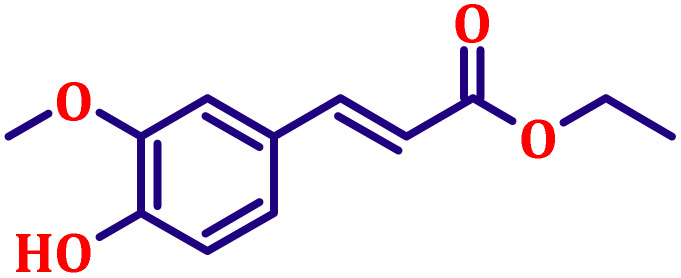	62
13	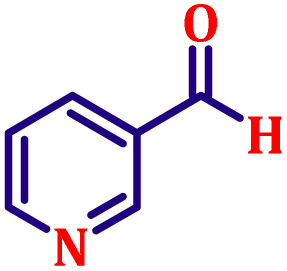	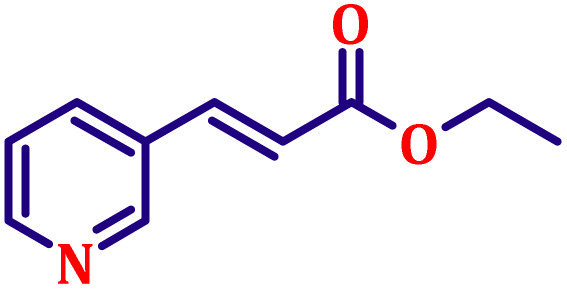	80
14	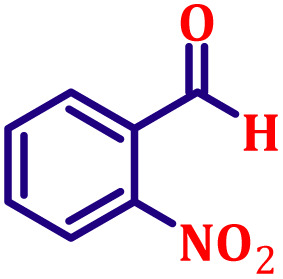	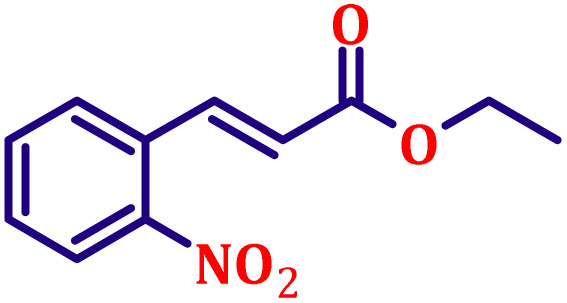	72
15	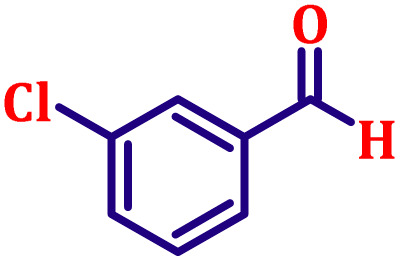	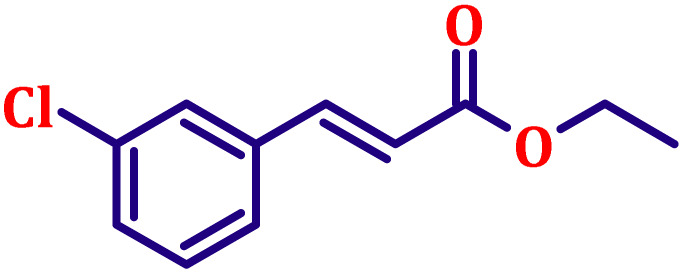	85

a Reaction conditions: substituted benzaldehyde (4.72 mmol), ethyl bromoacetate (4.72 mmol), PPh_3_ (4.72 mmol), catalyst TN3 (350) (25 mol%), DMF (5 mL), RT, 16 h.

b Isolated yield.

### 3.9. Spectroscopic data of some of the representative compounds

#### 3.9.1. Ethyl (*E*)-3-(4-chlorophenyl)acrylate (entry 1, Table 6)

Light yellow, clear liquid; b.p. 138 °C. ^1^H NMR (500 MHz, CDCl_3_) *δ* 7.54 (d, *J* = 16.0 Hz, 1H), 7.37 (d, *J* = 8.5 Hz, 2H), 7.27 (d, *J* = 8.5 Hz, 2H), 6.31 (d, *J* = 16.0 Hz, 1H), 4.17 (q, *J* = 7.0 Hz, 2H), 1.25 (t, *J* = 7.0 Hz, 3H). ^13^C NMR (126 MHz, CDCl_3_) *δ* 166.7, 143.1, 136.1, 133.0, 131.2, 128.2, 118.9, 60.7, 14.4.

#### 3.9.2. Ethyl (*E*)-3-(4-methoxyphenyl)acrylate (entry 2, Table 6)

White crystalline powder; m.p. 52 °C. ^1^H NMR (500 MHz, CDCl_3_) *δ* 7.55 (d, *J* = 16.0 Hz, 1H), 7.39 (d, *J* = 8.5 Hz, 2H), 6.82 (d, *J* = 8.5 Hz, 2H), 6.22 (d, *J* = 16.0 Hz, 1H), 4.16 (q, *J* = 7.5 Hz, 2H), 3.76 (s, 3H), 1.24 (t, *J* = 7.5 Hz, 3H). ^13^C NMR (126 MHz, CDCl_3_) *δ* 167.4, 161.3, 144.2, 129.7, 127.2, 115.8, 114.4, 60.4, 55.4, 14.4.

#### 3.9.3. Ethyl (*E*)-3-(4-fluorophenyl)acrylate (entry 3, Table 6)

Colourless liquid; b.p. 288 °C. ^1^H NMR (500 MHz, CDCl_3_) *δ* 7.56 (d, *J* = 16.0 Hz, 1H), 7.43–7.47 (m, 2H), 6.98–7.03 (m, 2H), 6.27 (d, *J* = 16.0 Hz, 1H), 4.17 (q, *J* = 7.0 Hz, 2H), 1.25 (t, *J* = 7.0 Hz, 3H). ^13^C NMR (126 MHz, CDCl_3_) *δ* 166.9, 162.9, 143.3, 130.8, 129.9, 118.1, 116.0, 60.6, 14.3.

#### 3.9.4. Ethyl (*E*)-3-(2-hydroxyphenyl)acrylate (entry 4, Table 6)

Off-white crystalline powder; m.p. 86 °C. ^1^H NMR (500 MHz, CDCl_3_) *δ* 7.92 (d, *J* = 16.0 Hz, 1H), 7.39 (dd, *J* = 7.5, 1.3 Hz, 1H), 7.14–7.19 (m, 1H), 6.83 (t, *J* = 7.5 Hz, 1H), 6.76 (d, *J* = 8.0 Hz, 1H), 6.52 (d, *J* = 16.0 Hz, 1H), 4.19 (q, *J* = 7.0 Hz, 2H), 1.26 (t, *J* = 7.0 Hz, 3H). ^13^C NMR (126 MHz, CDCl_3_) *δ* 168.5, 157.1, 140.1, 131.3, 129.2, 121.9, 120.9, 118.9, 116.3, 60.5, 14.4.

#### 3.9.5. Ethyl (*E*)-3-(2-chlorophenyl)acrylate (entry 5, Table 6)

Pale yellow liquid; b.p. 305 °C. ^1^H NMR (500 MHz, CDCl_3_) *δ* 8.00 (d, *J* = 16.0 Hz, 1H), 7.54 (dd, *J* = 7.5, 2.0 Hz, 1H), 7.34 (dd, *J* = 7.5, 2.0 Hz, 1H), 7.15–7.26 (m, 2H), 6.34 (d, *J* = 16.0 Hz, 1H), 4.19 (q, *J* = 7.0 Hz, 2H), 1.26 (t, *J* = 7.0 Hz, 3H). ^13^C NMR (126 MHz, CDCl_3_) *δ* 166.5, 140.4, 134.9, 132.8, 130.2, 129.1, 127.1, 122.1, 121.0, 60.7, 14.3.

#### 3.9.6. Ethyl (*E*)-3-(3-methoxyphenyl)acrylate (entry 6, Table 6)

Pale yellow liquid; b.p. 145 °C. ^1^H NMR (500 MHz, CDCl_3_) *δ* 7.56 (d, *J* = 16.0 Hz, 1H), 7.16–7.24 (m, 1H), 7.04 (d, *J* = 7.8 Hz, 1H), 6.97 (t, *J* = 2.0 Hz, 1H), 6.85 (dd, *J* = 8.0 Hz, 1H), 6.34 (d, *J* = 16.0 Hz, 1H), 4.17 (q, *J* = 7.0 Hz, 2H), 3.86 (s, 3H), 1.25 (t, *J* = 7.0 Hz, 3H). ^13^C NMR (126 MHz, CDCl_3_) *δ* 167.0, 159.9, 144.5, 135.9, 129.9, 120.8, 118.6, 116.1, 112.9, 60.5, 55.3, 14.3.

#### 3.9.7. Ethyl (*E*)-3-(3,4-dimethoxyphenyl)acrylate (entry 7, Table 6)

White crystalline powder; m.p. 52 °C. ^1^H NMR (500 MHz, CDCl_3_) *δ* 7.54 (d, *J* = 16.0 Hz, 1H), 7.02 (dd, *J* = 8.0, 2.0 Hz, 1H), 6.98 (d, *J* = 2.0 Hz, 1H), 6.78 (d, *J* = 8.0 Hz, 1H), 6.22 (d, *J* = 16.0 Hz, 1H), 4.16 (q, *J* = 7.0 Hz, 2H), 3.84 (s, 6H), 1.25 (t, *J* = 7.0 Hz, 3H). ^13^C NMR (126 MHz, CDCl_3_) *δ* 167.3, 151.2, 149.2, 144.5, 127.5, 122.5, 116.0, 111.1, 109.6, 60.3, 55.9, 14.4.

#### 3.9.8. Ethyl (*E*)-3-(2,4-dichlorophenyl)acrylate (entry 8, Table 6)

White crystalline powder; m.p. 85 °C. ^1^H NMR (500 MHz, CDCl_3_) *δ* 7.91 (d, *J* = 16.0 Hz, 1H), 7.46 (d, *J* = 8.5 Hz, 1H), 7.36 (d, *J* = 2.0 Hz, 1H), 7.18–7.20 (m, 1H), 6.32 (d, *J* = 16.0 Hz, 1H), 4.18 (q, *J* = 7.0 Hz, 2H), 1.25 (t, *J* = 7.0 Hz, 3H). ^13^C NMR (126 MHz, CDCl_3_) *δ* 166.2, 139.1, 136.3, 135.5, 131.4, 130.0, 128.4, 127.5, 121.4, 60.7, 14.2.

#### 3.9.9. Ethyl (*E*)-3-(4-nitrophenyl)acrylate (entry 9, Table 6)

Off-white crystalline powder; m.p. 138 °C. ^1^H NMR (500 MHz, CDCl_3_) *δ* 8.17 (d, *J* = 8.5 Hz, 2H), 7.62 (d, *J* = 16.0 Hz, 1H), 7.59 (d, *J* = 8.5 Hz, 2H), 6.47 (d, *J* = 16.0 Hz, 1H), 4.20 (q, *J* = 7.0 Hz, 2H), 1.27 (t, *J* = 7.0 Hz, 3H). ^13^C NMR (126 MHz, CDCl_3_) *δ* 166.0, 148.5, 141.6, 140.6, 128.6, 124.2, 122.6, 61.0, 14.3.

#### 3.9.10. Ethyl (*E*)-cinnamate (entry 10, Table 6)

Pale yellow liquid; b.p. 272 °C. ^1^H NMR (500 MHz, CDCl_3_) *δ* 7.60 (d, *J* = 16.0 Hz, 1H), 7.44 (dd, *J* = 8.0, 2.0 Hz, 2H), 7.29–7.33 (m, 3H), 6.35 (d, *J* = 16.0 Hz, 1H), 4.17 (q, *J* = 7.0 Hz, 2H), 1.25 (t, *J* = 7.0 Hz, 3H). ^13^C NMR (126 MHz, CDCl_3_) *δ* 167.0, 144.6, 134.5, 130.2, 128.9, 128.1, 118.3, 60.5, 14.3.

#### 3.9.11. Ethyl (*E*)-3-(4-hydroxyphenyl)acrylate (entry 11, Table 6)

White crystalline powder; m.p. 48 °C. ^1^H NMR (500 MHz, CDCl_3_) *δ* 7.54 (d, *J* = 16.0 Hz, 1H), 7.39 (d, *J* = 8.5 Hz, 2H), 6.82 (d, *J* = 8.5 Hz, 2H), 6.23 (d, *J* = 16.0 Hz, 1H), 4.57 (s, 1H), 4.16 (q, *J* = 7.0 Hz, 2H), 1.21 (t, *J* = 7.0 Hz, 3H). ^13^C NMR (126 MHz, CDCl_3_) *δ* 168.5, 159.4, 143.9, 129.7, 128.3, 116.5, 115.0, 60.4, 14.2.

## 4. Conclusion

In conclusion, various nanocrystalline nitrogen-doped TiO_2_ (N-TiO_2_) materials were successfully synthesized *via* a sol–gel method using triethylamine as the nitrogen source. Out of all nanocrystalline materials TN3 nanostructured material exhibited excellent heterogeneous catalytic performance in the one-pot Wittig reaction, affording α,β-unsaturated esters with high *E*-stereoselectivity and remarkable yields. The developed protocol is simple, economical, and environmentally benign, operating under mild reaction conditions with easy product separation and catalyst recyclability. These findings highlight N-TiO_2_ as an efficient and sustainable alternative catalyst for olefination reactions, demonstrating its potential applicability in fine chemical and pharmaceutical synthesis.

## Author contributions

Rohinee D. Hoval carried out the experimental work, including material synthesis, characterization, and catalytic studies, and contributed to data analysis and manuscript drafting. Santosh T. Shinde conceived and supervised the overall research work, designed the experiments, interpreted the results, and finalized the manuscript. Anandrao A. Kale and Nanasaheb S. Gaikwad assisted in experimental investigations and analytical data interpretation. Digambar B. Bankar contributed to spectroscopic analysis and discussion of results. Nitin M. Thorat supported materials characterization and provided technical inputs related to instrumentation. Ramesh B. Gawade supported in reaction optimization and experimental validation. Kaluram G. Kanade contributed to critical review, data interpretation, and improvement of the manuscript. Dinesh P. Amalnerkar provided overall guidance, critical revision of the manuscript, and final approval of the version to be published. All authors have read and approved the final manuscript.

## Conflicts of interest

There are no conflicts to declare.

## Supplementary Material

RA-016-D5RA09170E-s001

## Data Availability

All data generated or analyzed during this study are included in this published article and its supplementary information (SI) files. Supplementary information: ^1^H NMR and ^13^C NMR spectra of the representative synthesized compounds, provided for structural confirmation. See DOI: https://doi.org/10.1039/d5ra09170e.
