# Supplementary material for: N-doped TiO2 nanocatalyst for one-pot synthesis of α,β-unsaturated esters via the Wittig reaction: a highly efficient and recyclable heterogeneous system
Source: RSC Adv. 2026 Feb 26;16(12):11294–305. doi: 10.1039/d5ra09170e (PMC12937064; doi:10.1039/d5ra09170e)
Supplement: RA-016-D5RA09170E-s001 [file RA-016-D5RA09170E-s001.pdf]

Supplementary Information (SI) for RSC Advances.
This journal is © The Royal Society of Chemistry 2026

Electronic Supplementary Information (ESI)

N-Doped TiO_2 Nanocatalyst for One-Pot Synthesis of α,β-Unsaturated Esters via the Wittig Reaction: A Highly Efficient and Recyclable Heterogeneous System

Rohinee D. Hoval[1], Santosh T. Shinde[1]*, Anandrao A. Kale[1], Nanasaheb S. Gaikwad[1], Digambar B. Bankar[3], Nitin M. Thorat[4], Ramesh B. Gawade[1], Kaluram G. Kanade[5], Dinesh P. Amalnerkar[2]

[1]*Post graduate Department and Research Centre of Chemistry, Annasaheb Awate College Manchar-410503, India.*

[2]*Pimpri Chinchwad University, Pune -412106, India.*

[3]*Post Graduate Department of Chemistry and Research Centre, R. B. Narayanrao Borawake College, Shrirampur (Autonomous), 413709, India.*

[4]*Post Graduate Department of Chemistry, and Research Centre, Maharaja Jivajirao Shinde Mahavidyalaya, Shrigonda, Ahilyanagar-413701, India.*

[5]*Rajmata Jijau Shikshan Prasarak Mandal's Arts, Commerce & Science College, Landewadi, Bhosari, Pune-411039, India.*

Corresponding Autor: Dr. Santosh T. Shinde, drsantoshinde@gmail.com

---

Supporting Data: ^{1}H-NMR and ^{13}C-NMR spectra of as-synthesized of compounds.

1) Nuclear Magnetic Resonance (NMR) of as-synthesized α,β-Unsaturated Esters (Table 6, entries 1 to 11):

i) Ethyl (E)-3-(4-chlorophenyl) acrylate (Entry 1, Table 6):

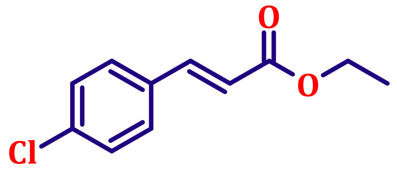

Light yellow, clear liquid; b.p. 138 °C. ^{1}H NMR (500 MHz, $CDCl_3$) δ 7.54 (d, *J* = 16.0 Hz, 1H), 7.37 (d, *J* = 8.5 Hz, 2H), 7.27 (d, *J* = 8.5 Hz, 2H), 6.31 (d, *J* = 16.0 Hz, 1H), 4.17 (q, *J* = 7.0 Hz, 2H), 1.25 (t, *J* = 7.0 Hz, 3H). ^{13}C NMR (126 MHz, $CDCl_3$) δ 166.7, 143.1, 136.1, 133.0, 131.2, 128.2, 118.9, 60.7, 14.4.

ii) Ethyl (E)-3-(4-methoxyphenyl) acrylate (Entry 2, Table 6):

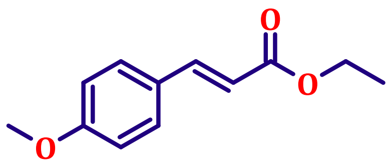

White crystalline powder; m.p. 52 °C. ^{1}H NMR (500 MHz, $CDCl_3$) δ 7.55 (d, *J* = 16.0 Hz, 1H), 7.39 (d, *J* = 8.5 Hz, 2H), 6.82 (d, *J* = 8.5 Hz, 2H), 6.22 (d, *J* = 16.0 Hz, 1H), 4.16 (q, *J* = 7.5 Hz, 2H), 3.76 (s, 3H), 1.24 (t, *J* = 7.5 Hz, 3H). ^{13}C NMR (126 MHz, $CDCl_3$) δ 167.4, 161.3, 144.2, 129.7, 127.2, 115.8, 114.4, 60.4, 55.4, 14.4.

iii) Ethyl (E)-3-(4-fluorophenyl) acrylate (Entry 3, Table 6):

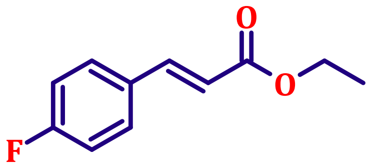

Colourless liquid; b.p. 288 °C. ^{1}H NMR (500 MHz, $CDCl_3$) δ 7.56 (d, *J* = 16.0 Hz, 1H), 7.43–7.47 (m, 2H), 6.98–7.03 (m, 2H), 6.27 (d, *J* = 16.0 Hz, 1H), 4.17 (q, *J* = 7.0 Hz, 2H), 1.25 (t, *J* = 7.0 Hz, 3H). ^{13}C NMR (126 MHz, $CDCl_3$) δ 166.9, 162.9, 143.3, 130.8, 129.9, 118.1, 116.0, 60.6, 14.3.

iv) Ethyl (E)-3-(2-hydroxyphenyl) acrylate (Entry 4, Table 6):

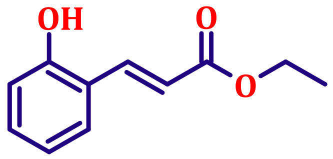

Off-white crystalline powder; m.p. 86 °C. ^{1}H NMR (500 MHz, $CDCl_3$) δ 7.92 (d, *J* = 16.0 Hz, 1H), 7.39 (dd, *J* = 7.5, 1.3 Hz, 1H), 7.14–7.19 (m, 1H), 6.83 (t, *J* = 7.5 Hz, 1H), 6.76 (d, *J* = 8.0 Hz, 1H), 6.52 (d, *J* = 16.0 Hz, 1H), 4.19 (q, *J* = 7.0 Hz, 2H), 1.26 (t, *J* = 7.0 Hz, 3H). ^{13}C NMR (126 MHz, $CDCl_3$) δ 168.5, 157.1, 140.1, 131.3, 129.2, 121.9, 120.9, 118.9, 116.3, 60.5, 14.4.

v) Ethyl (E)-3-(2-chlorophenyl) acrylate (Entry 5, Table 6):

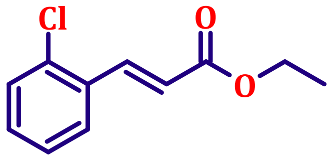

Pale yellow liquid; b.p. 305 °C. ^{1}H NMR (500 MHz, $CDCl_3$) δ 8.00 (d, *J* = 16.0 Hz, 1H), 7.54 (dd, *J* = 7.5, 2.0 Hz, 1H), 7.34 (dd, *J* = 7.5, 2.0 Hz, 1H), 7.15–7.26 (m, 2H), 6.34 (d, *J* = 16.0 Hz, 1H), 4.19 (q, *J* = 7.0 Hz, 2H), 1.26 (t, *J* = 7.0 Hz, 3H). ^{13}C NMR (126 MHz, $CDCl_3$) δ 166.5, 140.4, 134.9, 132.8, 130.2, 129.1, 127.1, 122.1, 121.0, 60.7, 14.3.

vi) Ethyl (E)-3-(3-methoxyphenyl) acrylate (Entry 6, Table 6):

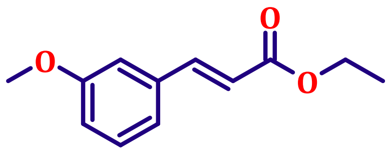

Pale yellow liquid; b.p. 145 °C. ^{1}H NMR (500 MHz, $CDCl_3$) δ 7.56 (d, *J* = 16.0 Hz, 1H), 7.16–7.24 (m, 1H), 7.04 (d, *J* = 7.8 Hz, 1H), 6.97 (t, *J* = 2.0 Hz, 1H), 6.85 (dd, *J* = 8.0 Hz, 1H), 6.34 (d, *J* = 16.0 Hz, 1H), 4.17 (q, *J* = 7.0 Hz, 2H), 3.86 (s, 3H), 1.25 (t, *J* = 7.0 Hz, 3H). ^{13}C NMR (126 MHz, $CDCl_3$) δ 167.0, 159.9, 144.5, 135.9, 129.9, 120.8, 118.6, 116.1, 112.9, 60.5, 55.3, 14.3.

vii) Ethyl (E)-3-(3,4-dimethoxyphenyl) acrylate (Entry 7, Table 6):

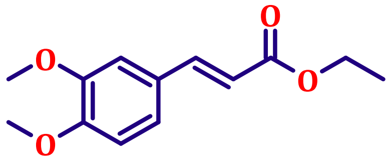

White crystalline powder; m.p. 52 °C. ^{1}H NMR (500 MHz, $CDCl_3$) δ 7.54 (d, *J* = 16.0 Hz, 1H), 7.02 (dd, *J* = 8.0, 2.0 Hz, 1H), 6.98 (d, *J* = 2.0 Hz, 1H), 6.78 (d, *J* = 8.0 Hz, 1H), 6.22 (d, *J* = 16.0 Hz, 1H), 4.16 (q, *J* = 7.0 Hz, 2H), 3.84 (s, 6H), 1.25 (t, *J* = 7.0 Hz, 3H). ^{13}C NMR (126 MHz, $CDCl_3$) δ 167.3, 151.2, 149.2, 144.5, 127.5, 122.5, 116.0, 111.1, 109.6, 60.3, 55.9, 14.4.

viii) Ethyl (E)-3-(2,4-dichlorophenyl) acrylate (Entry 8, Table 6):

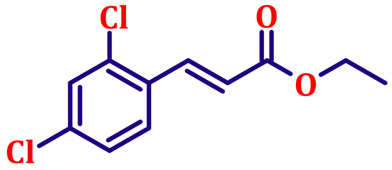

White crystalline powder; m.p. 85 °C. ^{1}H NMR (500 MHz, $CDCl_3$) δ 7.91 (d, *J* = 16.0 Hz, 1H), 7.46 (d, *J* = 8.5 Hz, 1H), 7.36 (d, *J* = 2.0 Hz, 1H), 7.18–7.20 (m, 1H), 6.32 (d, *J* = 16.0 Hz, 1H), 4.18 (q, *J* = 7.0 Hz, 2H), 1.25 (t, *J* = 7.0 Hz, 3H). ^{13}C NMR (126 MHz, $CDCl_3$) δ 166.2, 139.1, 136.3, 135.5, 131.4, 130.0, 128.4, 127.5, 121.4, 60.7, 14.2.

ix) Ethyl (E)-3-(4-nitrophenyl) acrylate (Entry 9, Table 6):

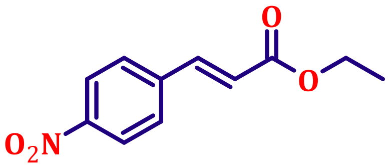

Off-white crystalline powder; m.p. 138 °C. ^{1}H NMR (500 MHz, $CDCl_3$) δ 8.17 (d, *J* = 8.5 Hz, 2H), 7.62 (d, *J* = 16.0 Hz, 1H), 7.59 (d, *J* = 8.5 Hz, 2H), 6.47 (d, *J* = 16.0 Hz, 1H), 4.20 (q, *J* = 7.0 Hz, 2H), 1.27 (t, *J* = 7.0 Hz, 3H). ^{13}C NMR (126 MHz, $CDCl_3$) δ 166.0, 148.5, 141.6, 140.6, 128.6, 124.2, 122.6, 61.0, 14.3.

x) Ethyl cinnamate (Entry 10, Table 6):

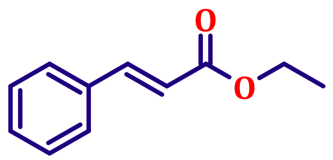

Pale yellow liquid; b.p. 272 °C. ^{1}H NMR (500 MHz, $CDCl_3$) δ 7.60 (d, *J* = 16.0 Hz, 1H), 7.44 (dd, *J* = 8.0, 2.0 Hz, 2H), 7.29–7.33 (m, 3H), 6.35 (d, *J* = 16.0 Hz, 1H), 4.17 (q, *J* = 7.0 Hz, 2H), 1.25 (t, *J* = 7.0 Hz, 3H). ^{13}C NMR (126 MHz, $CDCl_3$) δ 167.0, 144.6, 134.5, 130.2, 128.9, 128.1, 118.3, 60.5, 14.3.

xi) Ethyl (E)-3-(4-hydroxyphenyl) acrylate (Entry 11, Table 6):

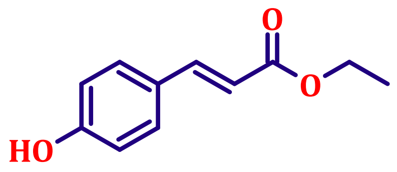

White crystalline powder; m.p. 48 °C. ^{1}H NMR (500 MHz, $CDCl_3$) δ 7.54 (d, *J* = 16.0 Hz, 1H), 7.39 (d, *J* = 8.5 Hz, 2H), 6.82 (d, *J* = 8.5 Hz, 2H), 6.23 (d, *J* = 16.0 Hz, 1H), 4.57 (s, 1H), 4.16 (q, *J* = 7.0 Hz, 2H), 1.21 (t, *J* = 7.0 Hz, 3H). ^{13}C NMR (126 MHz, $CDCl_3$) δ 168.5, 159.4, 143.9, 129.7, 128.3, 116.5, 115.0, 60.4, 14.2.

The structures of the synthesized representative compounds (Table 6, entries 1–11) were confirmed by ^{1}H NMR and ^{13}C NMR spectroscopy. The complete spectra, along with their expanded regions, are provided below. Information.

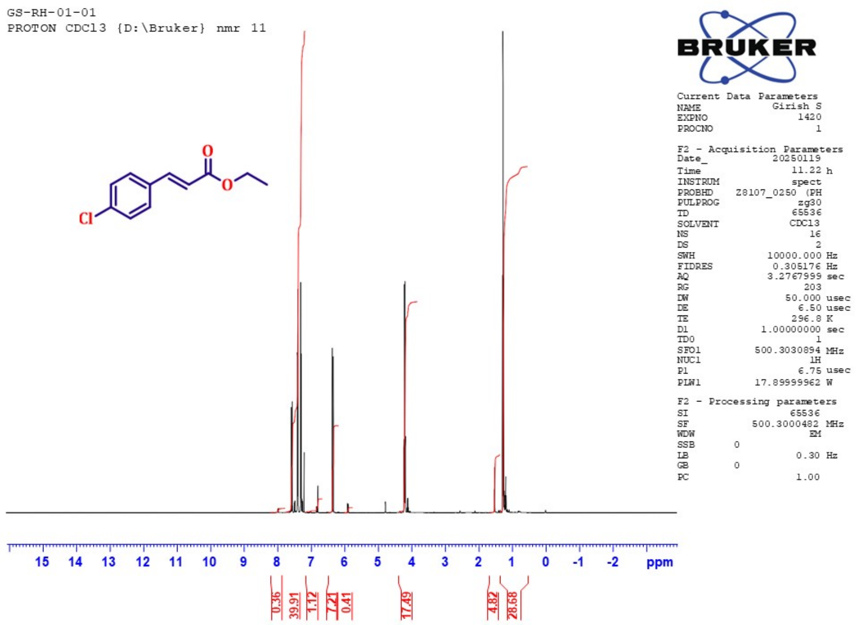

^{1}H-NMR spectrum of Ethyl *(E)*-3-(4-chlorophenyl)acrylate (Entry 1, Table 6)

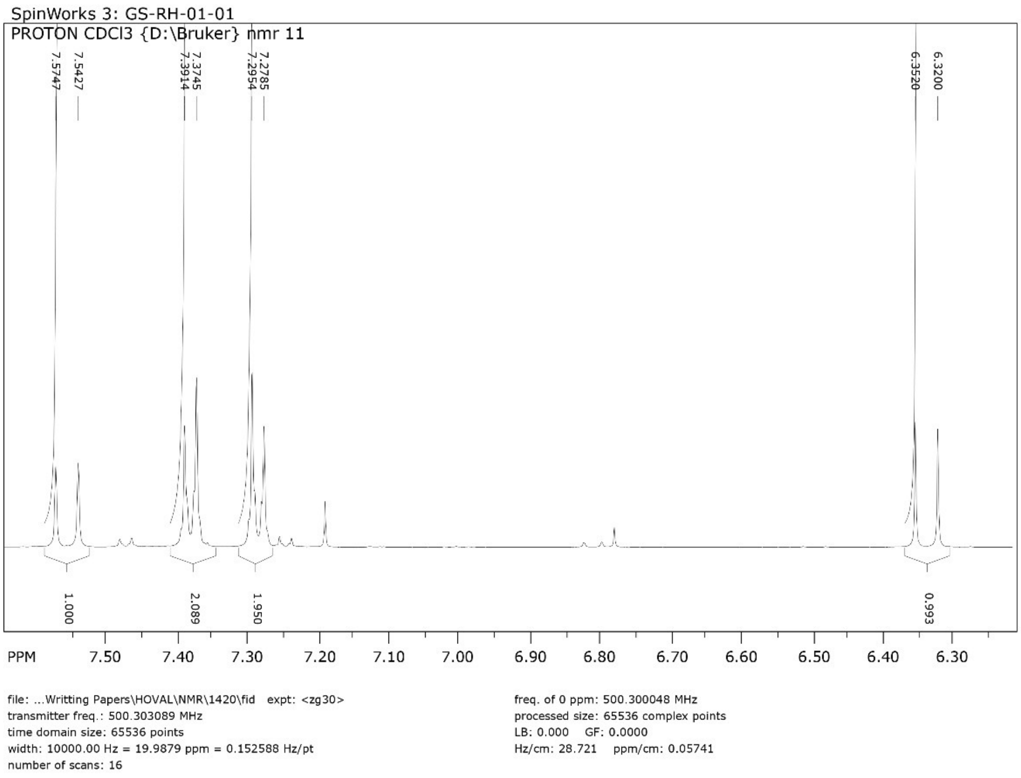

^{1}H-NMR spectrum of Ethyl *(E)*-3-(4-chlorophenyl)acrylate (Entry 1, Table 6, Expansion 1)

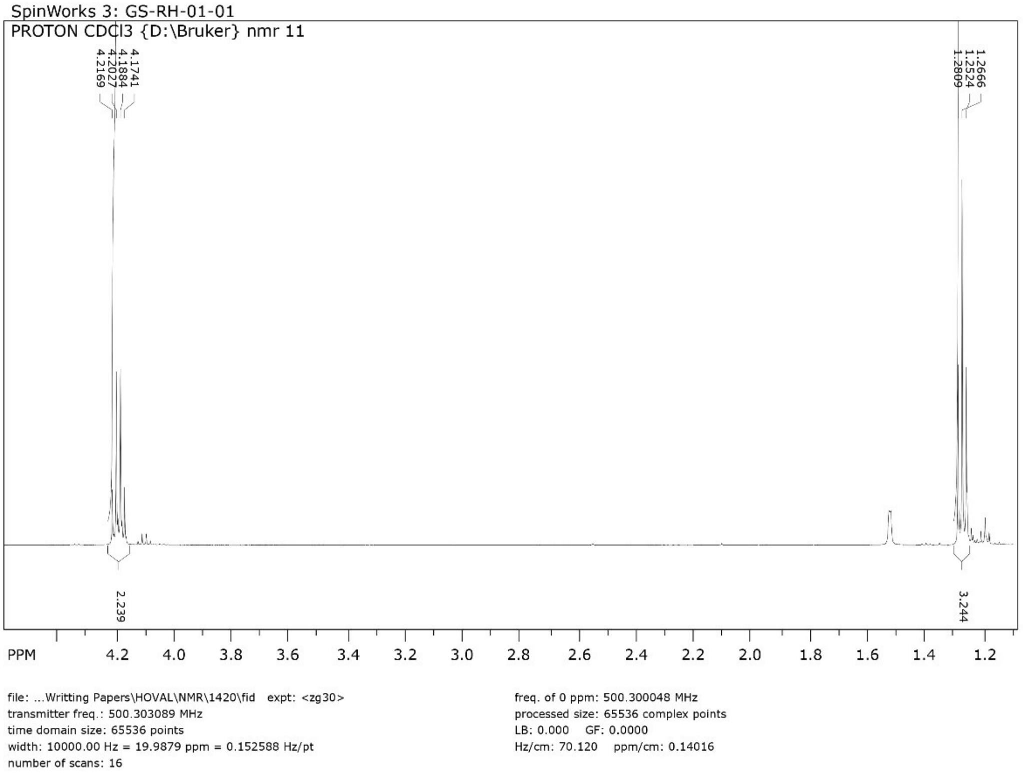

^{1}H-NMR spectrum of Ethyl *(E)*-3-(4-chlorophenyl)acrylate (Entry 1, Table 6, Expansion 2)

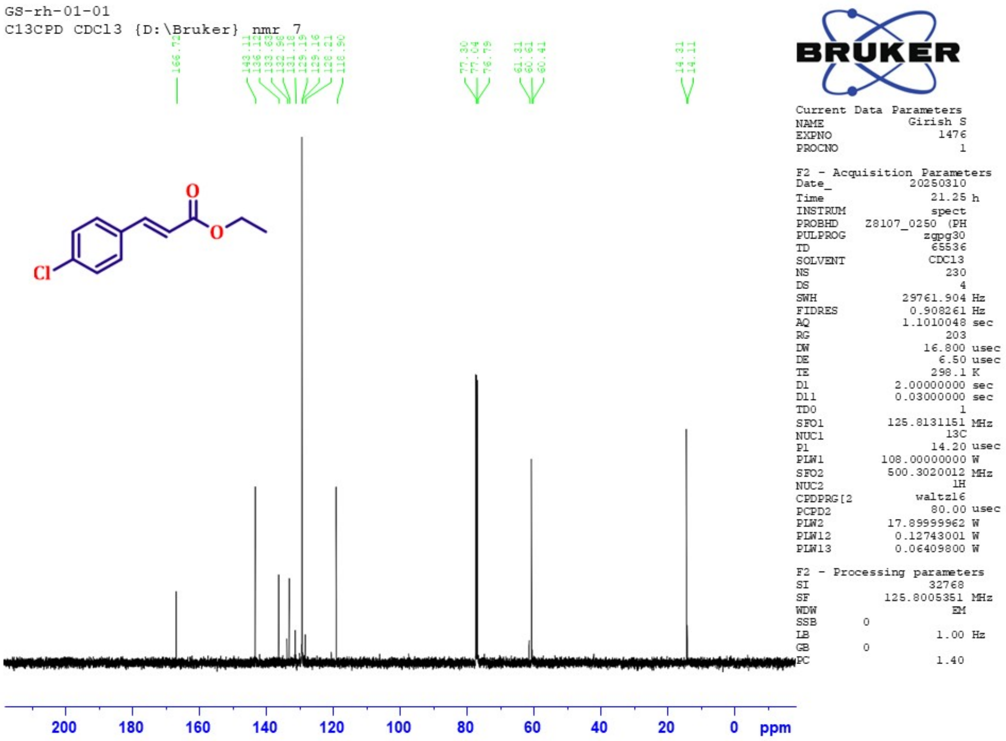

^{13}C-NMR spectrum of Ethyl *(E)*-3-(4-chlorophenyl)acrylate (Entry 1, Table 6)

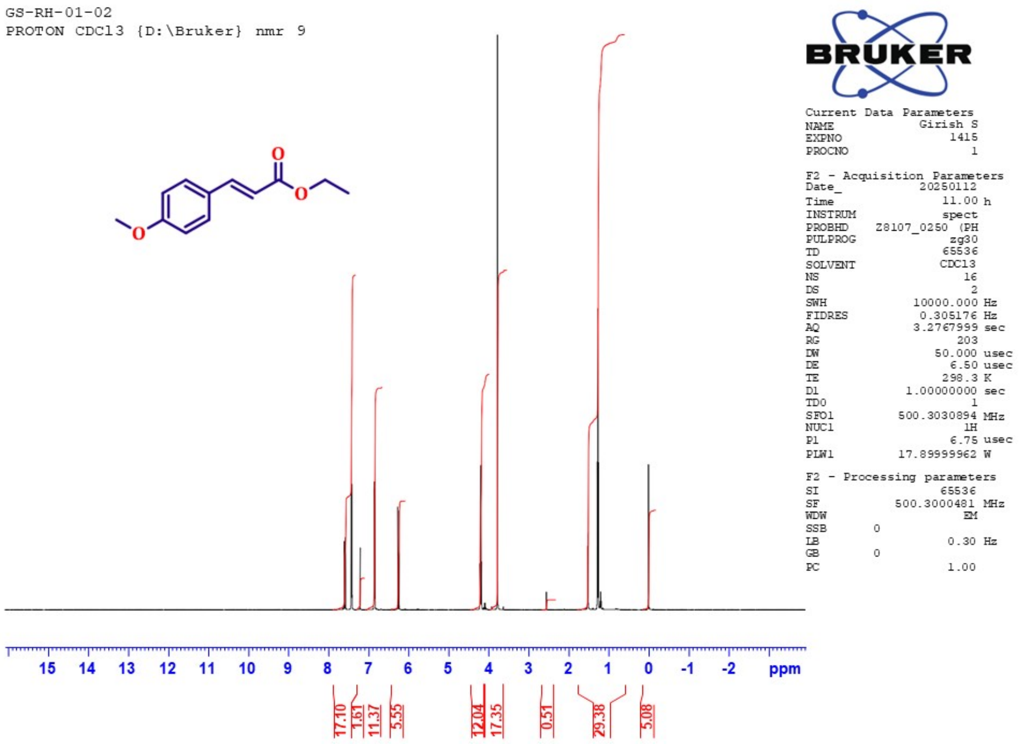

1H-NMR spectrum of Ethyl *(E)*-3-(4-methoxyphenyl)acrylate (Entry 2, Table 6)

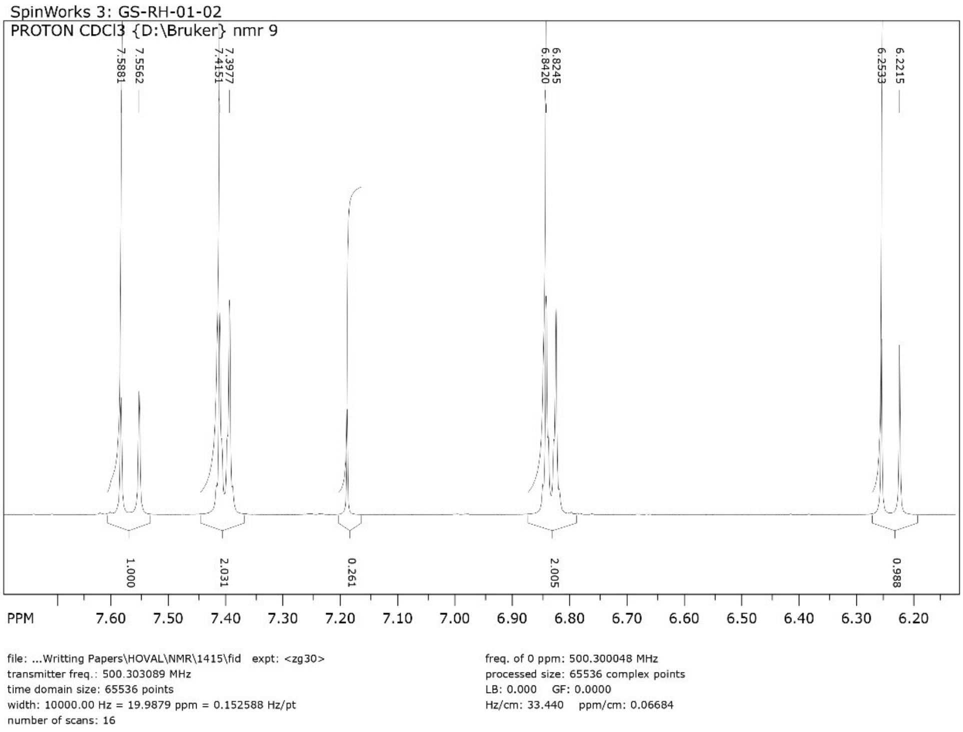

1H-NMR spectrum of Ethyl *(E)*-3-(4-methoxyphenyl)acrylate (Entry 2, Table 6, Expansion 1)

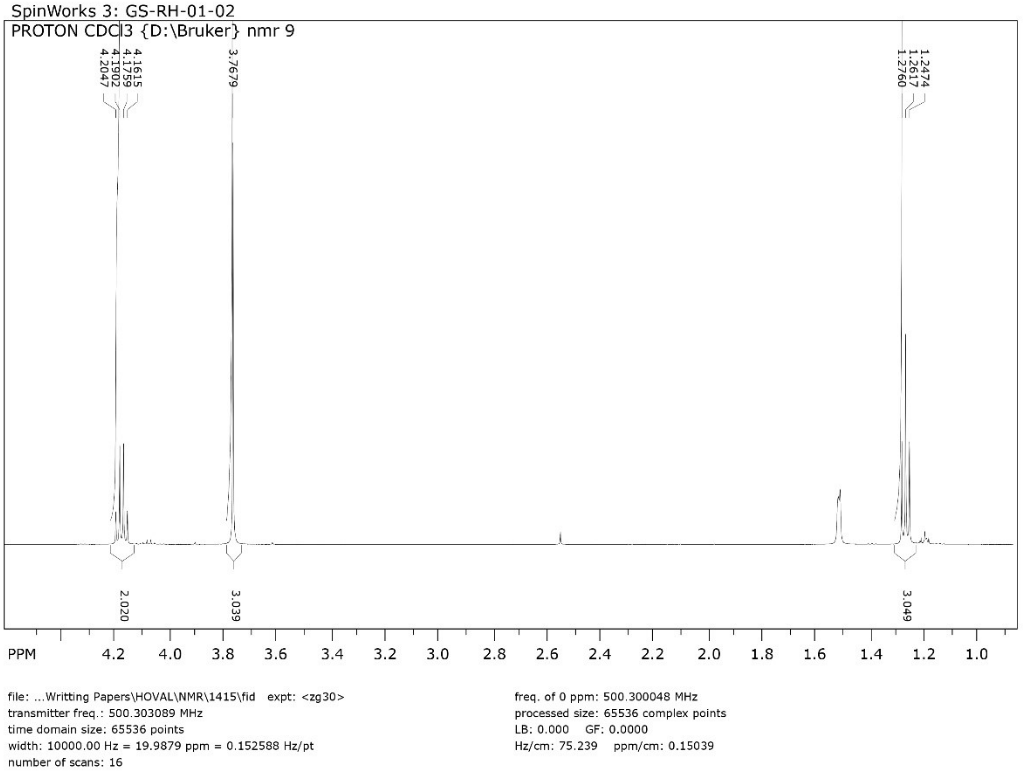

1H-NMR spectrum of Ethyl *(E)*-3-(4-methoxyphenyl)acrylate (Entry 2, Table 6, Expansion 2)

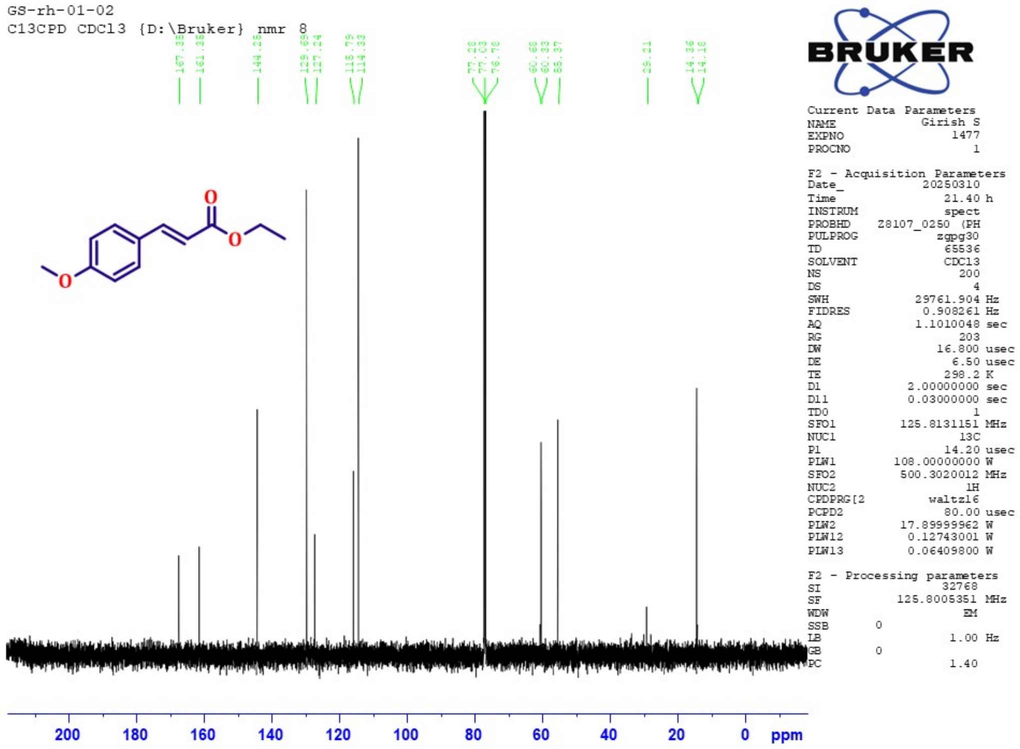

^{13}C NMR spectrum of Ethyl *(E)*-3-(4-methoxyphenyl)acrylate (Entry 2, Table 6)

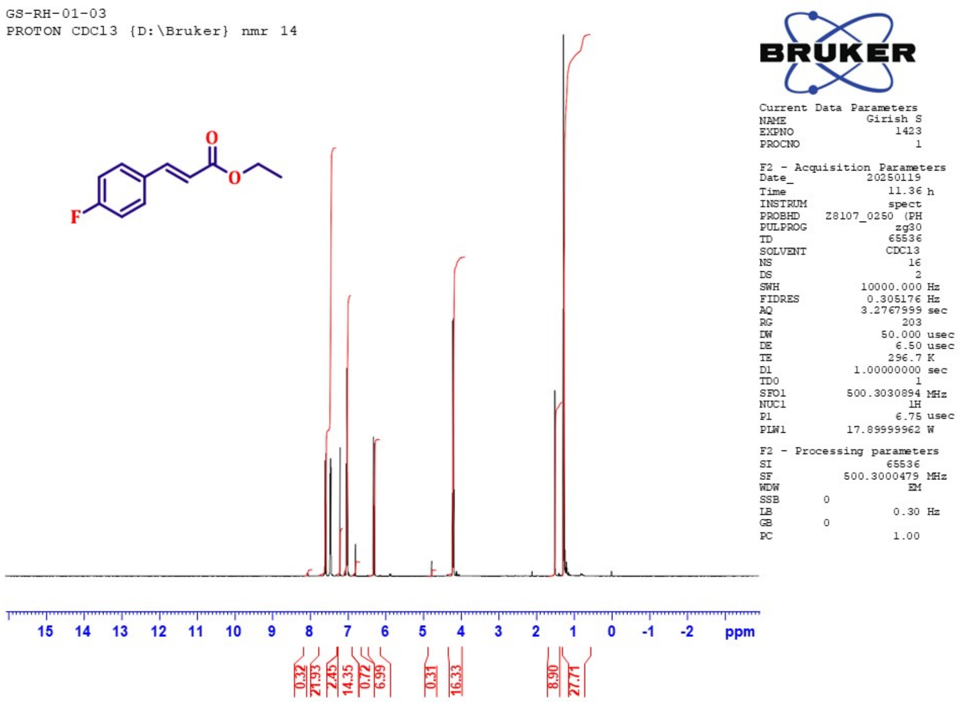

1H-NMR spectrum of Ethyl *(E)*-3-(4-fluorophenyl)acrylate (Entry 3, Table 6)

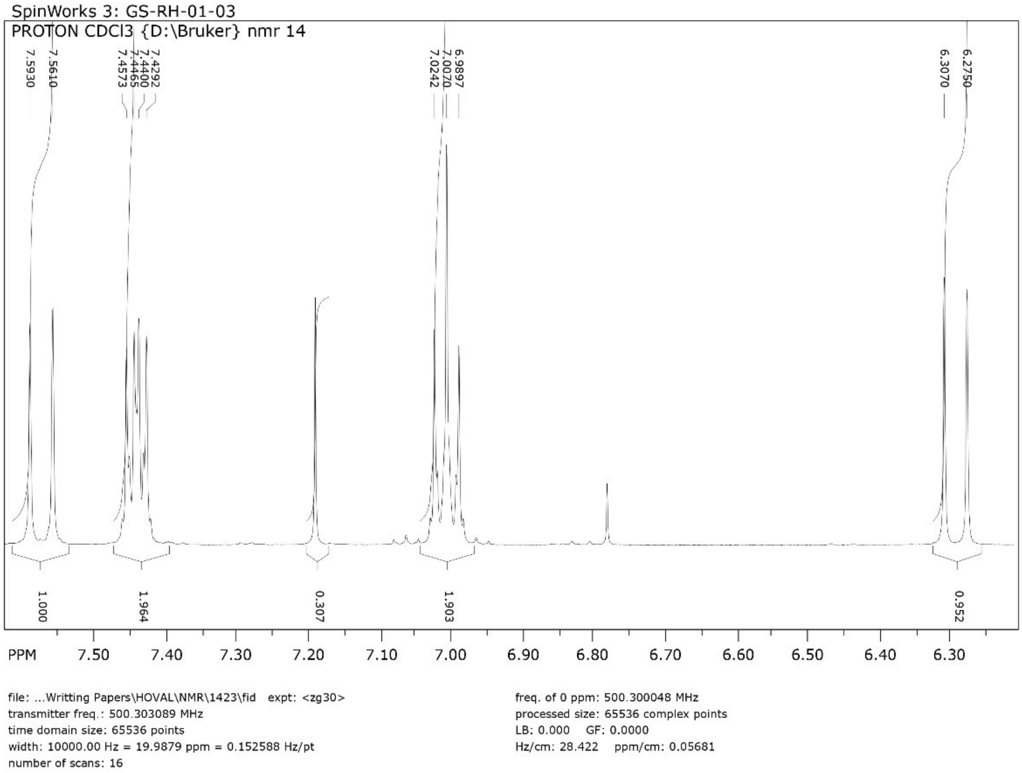

1H-NMR spectrum of Ethyl *(E)*-3-(4-fluorophenyl)acrylate (Entry 3, Table 6, Expansion 1)

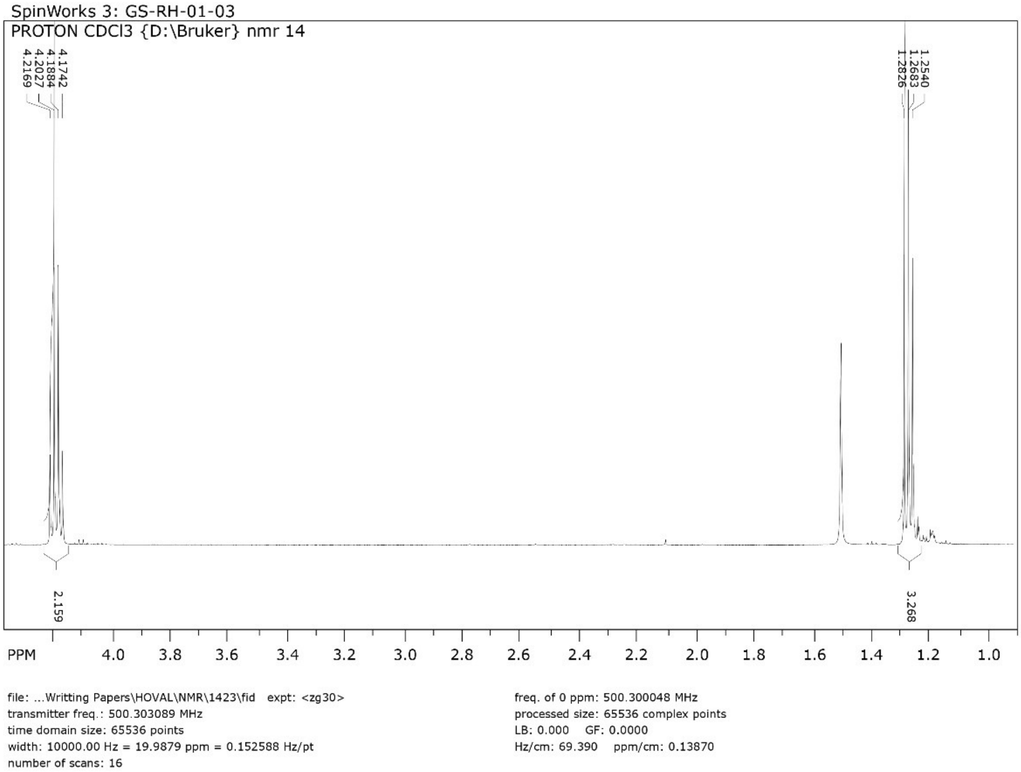

1H-NMR spectrum of Ethyl *(E)*-3-(4-fluorophenyl)acrylate (Entry 3, Table 6, Expansion 2)

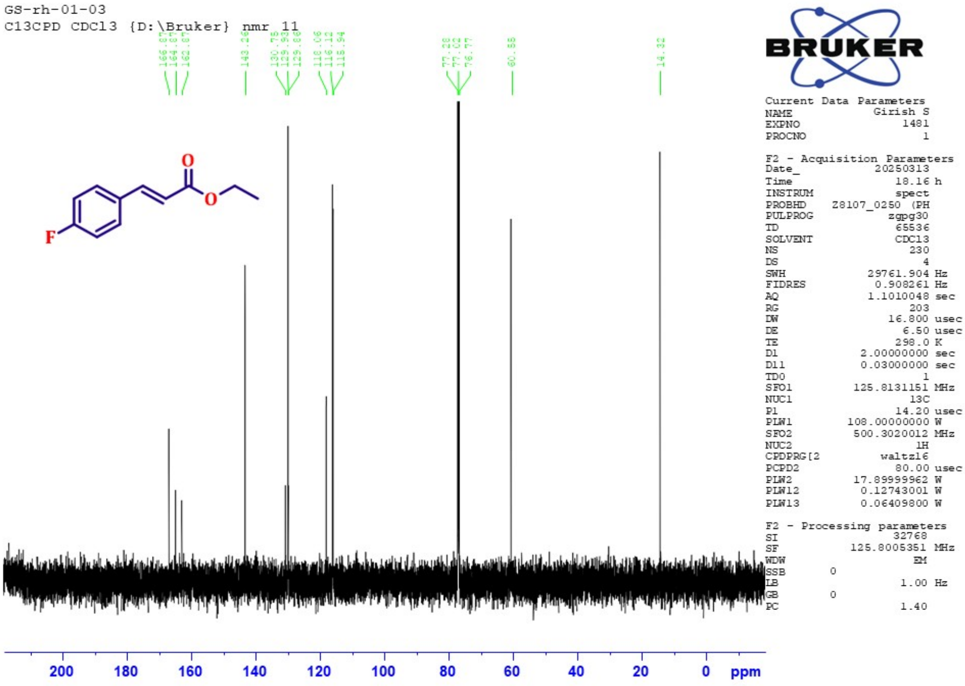

13C NMR spectrum of Ethyl *(E)*-3-(4-fluorophenyl)acrylate (Entry 3, Table 6)

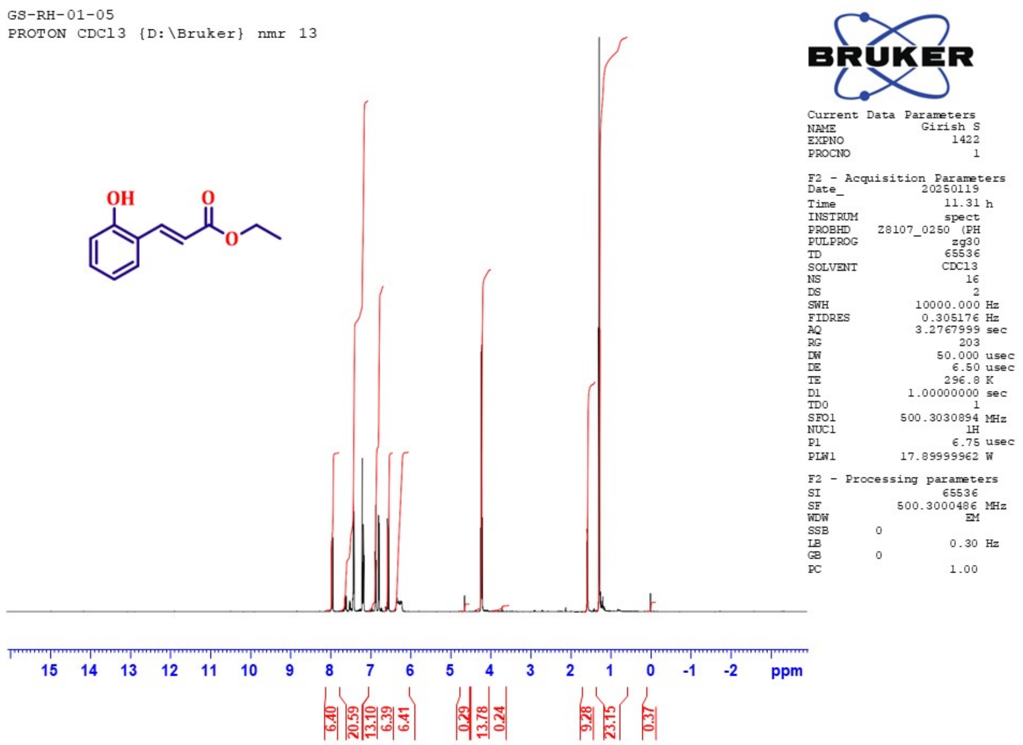

1H-NMR spectrum of Ethyl *(E)*-3-(2-hydroxyphenyl)acrylate (Entry 4, Table 6)

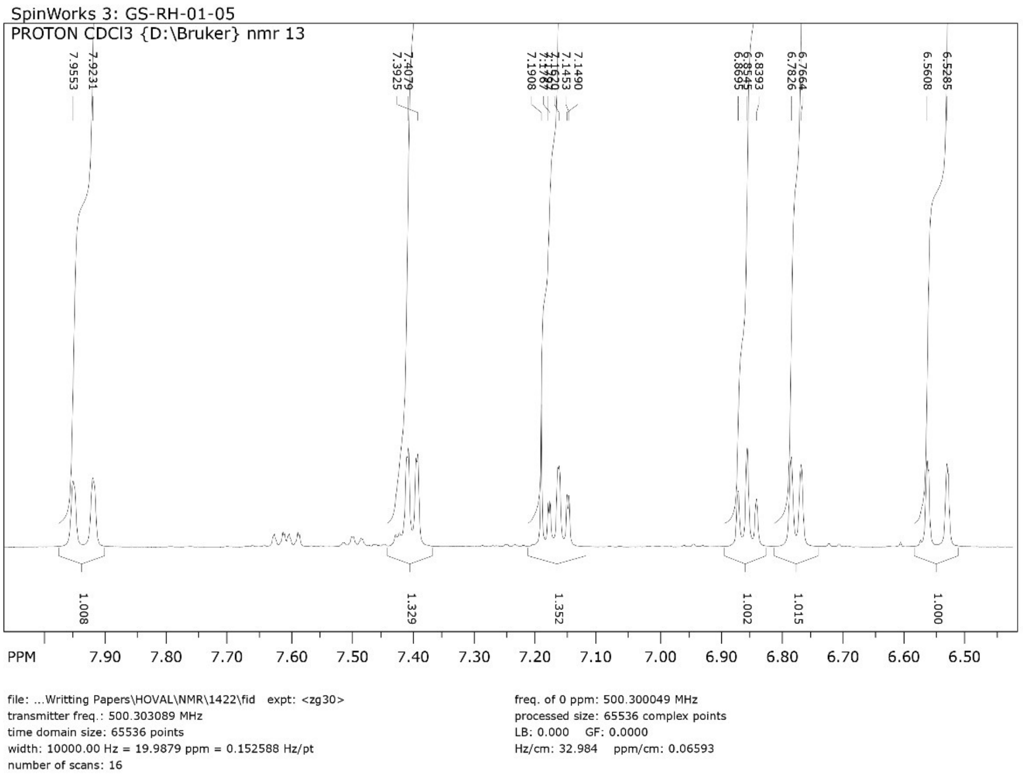

1H-NMR spectrum of Ethyl *(E)*-3-(2-hydroxyphenyl)acrylate (Entry 4, Table 6, Expansion 1)

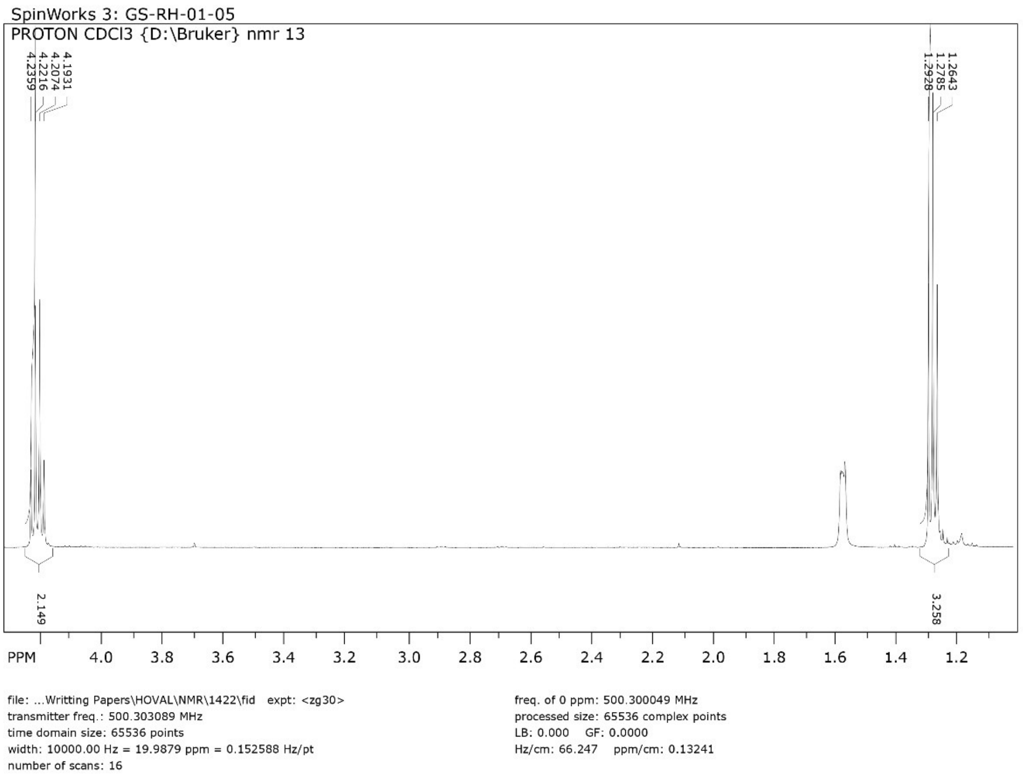

1H-NMR spectrum of Ethyl *(E)*-3-(2-hydroxyphenyl)acrylate (Entry 4, Table 6, Expansion 2)

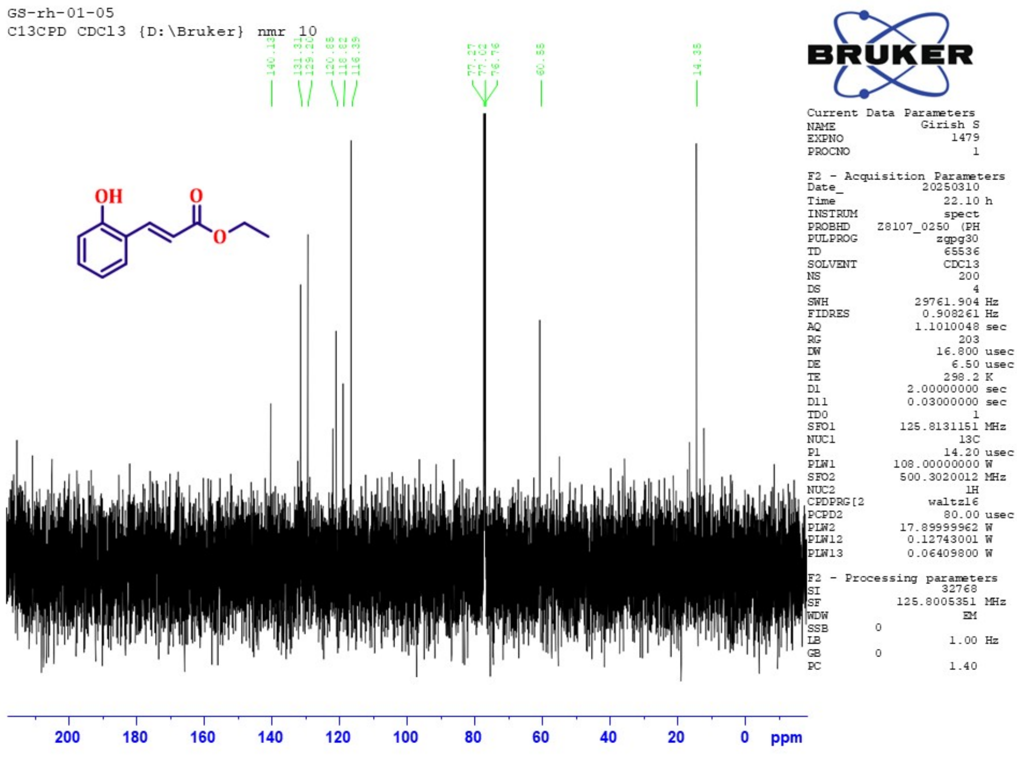

13C NMR spectrum of Ethyl *(E)*-3-(2-hydroxyphenyl)acrylate (Entry 4, Table 6)

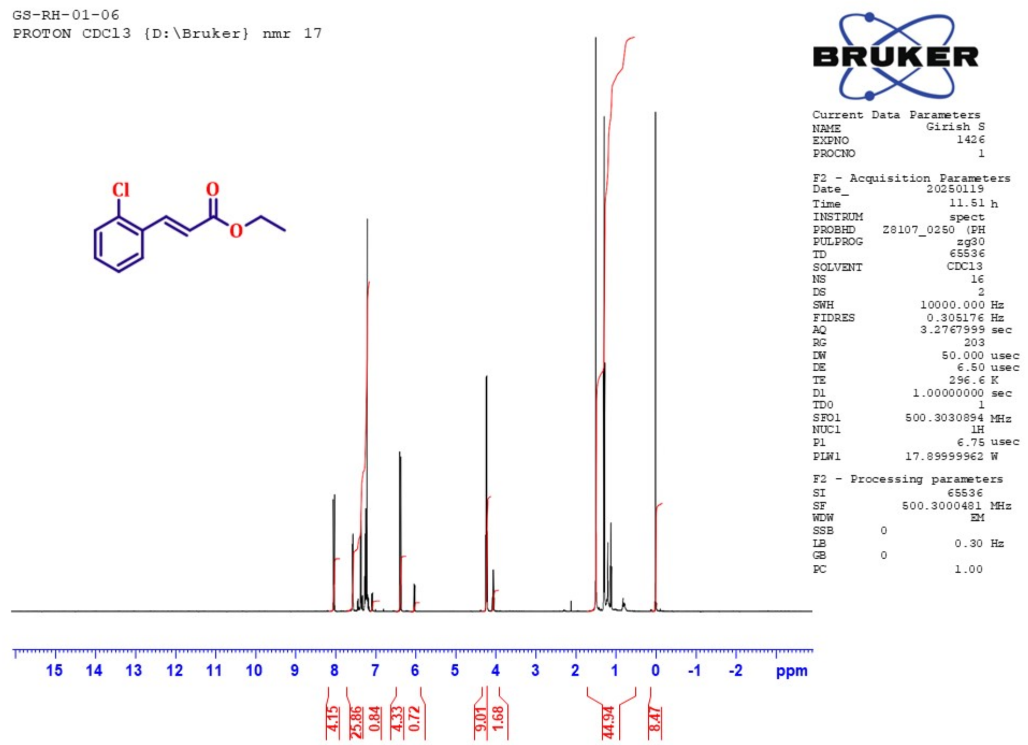

1H-NMR spectrum of Ethyl *(E)*-3-(2-chlorophenyl)acrylate (Entry 5, Table 6)

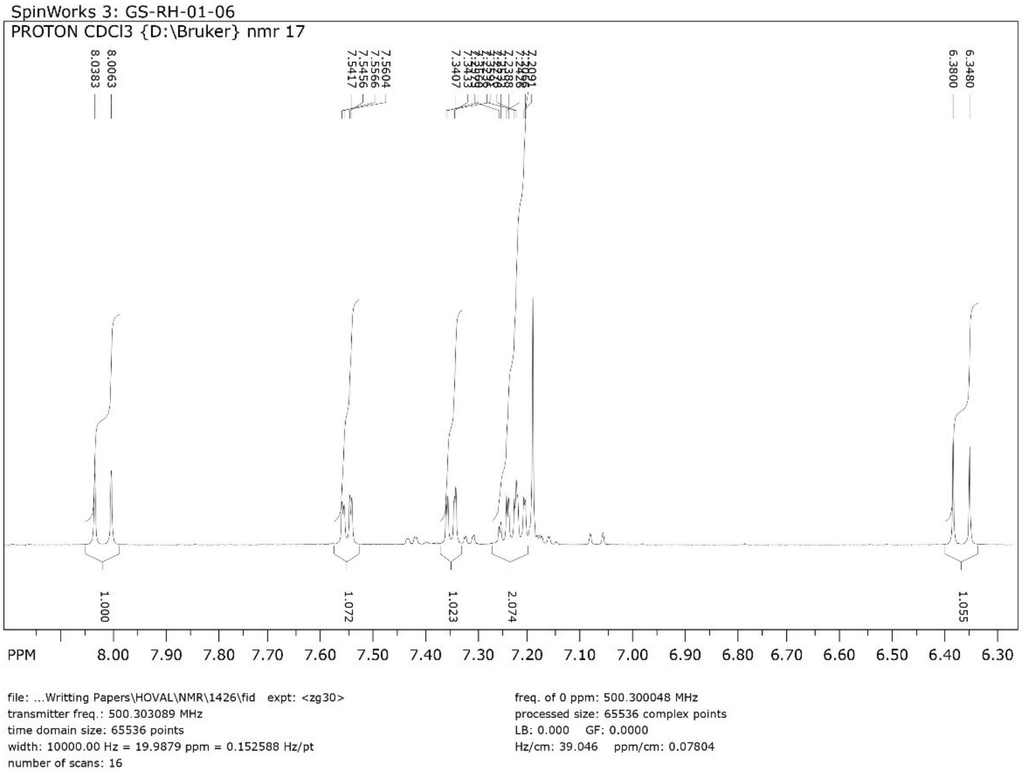

1H-NMR spectrum of Ethyl *(E)*-3-(2-chlorophenyl)acrylate (Entry 5, Table 6, Expansion 1)

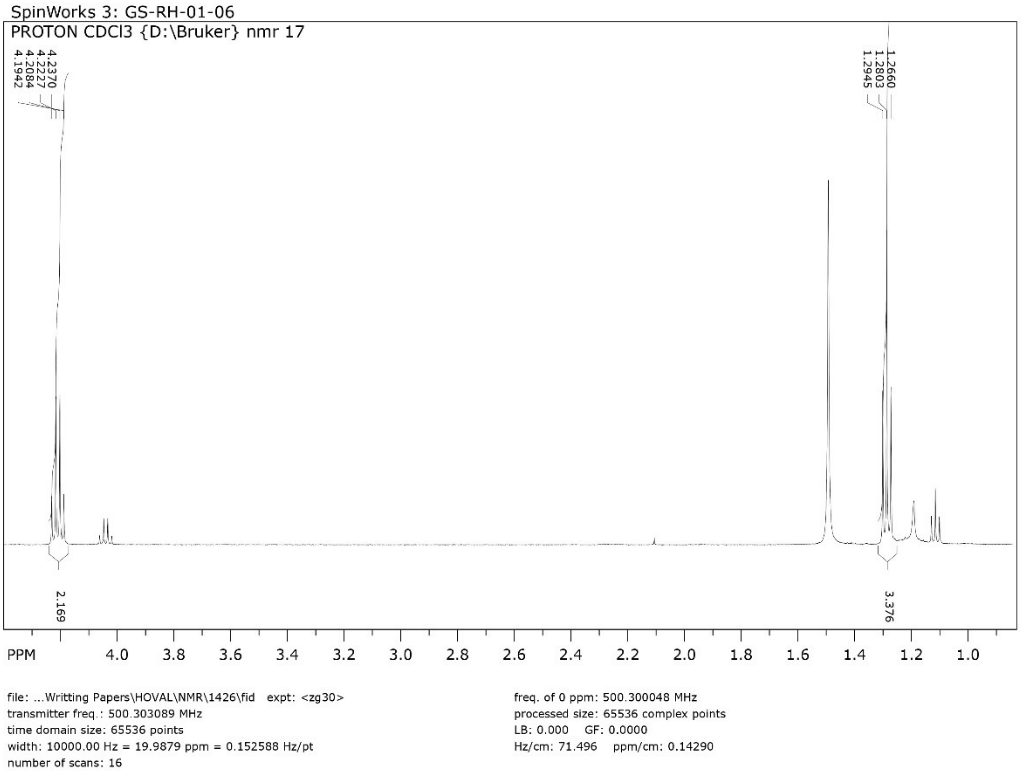

1H-NMR spectrum of Ethyl *(E)*-3-(2-chlorophenyl)acrylate (Entry 5, Table 6, Expansion 2)

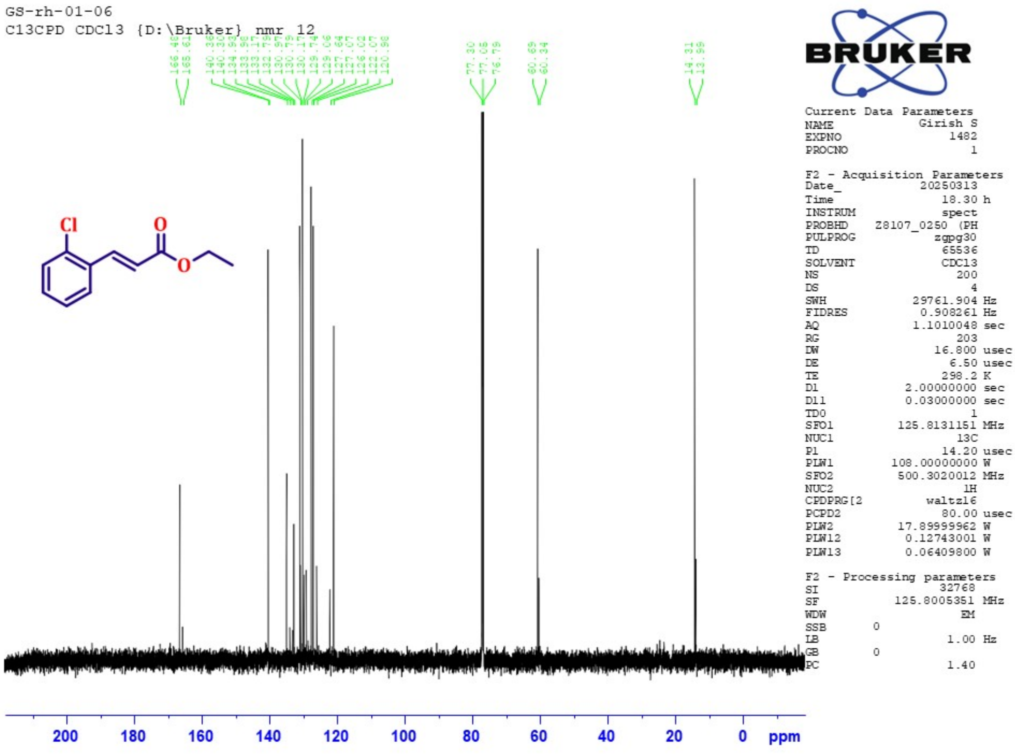

13C NMR spectrum of Ethyl *(E)*-3-(2-chlorophenyl)acrylate (Entry 5, Table 6)

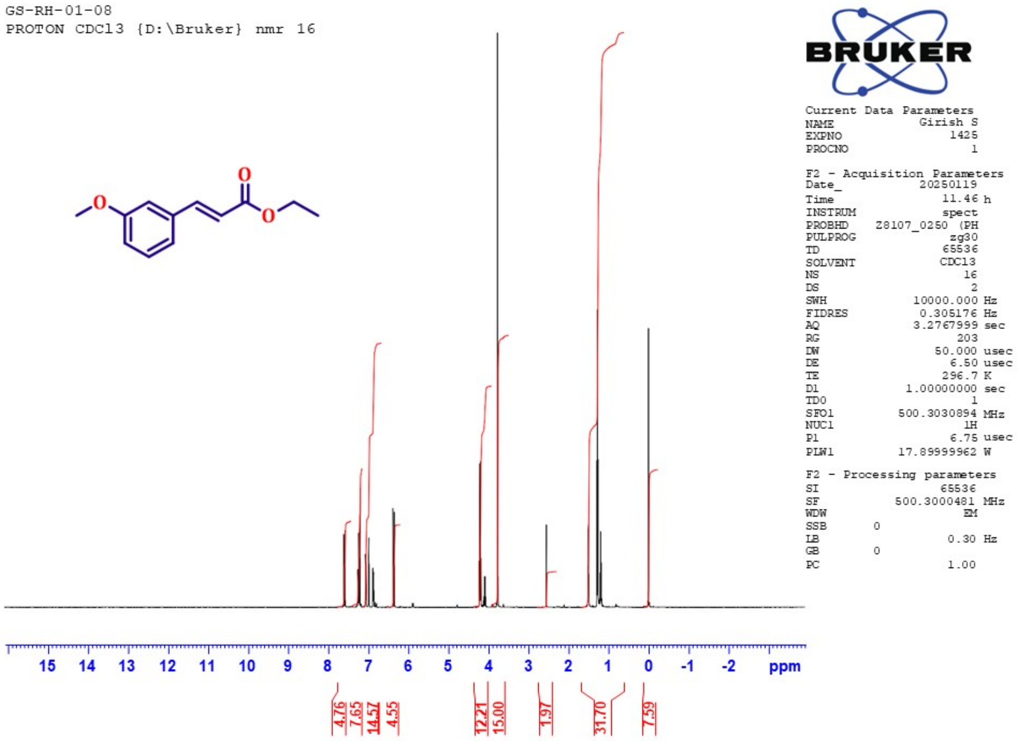

1H-NMR spectrum of Ethyl *(E)*-3-(3-methoxyphenyl)acrylate (Entry 6, Table 6)

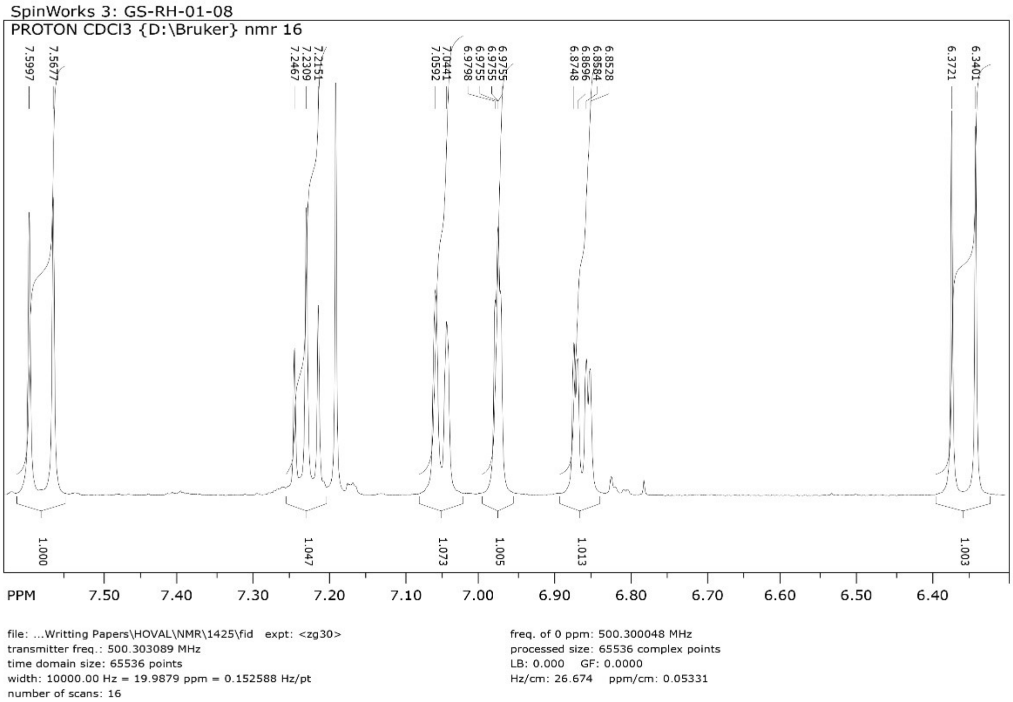

1H-NMR spectrum of Ethyl *(E)*-3-(3-methoxyphenyl)acrylate (Entry 6, Table 6, Expansion 1)

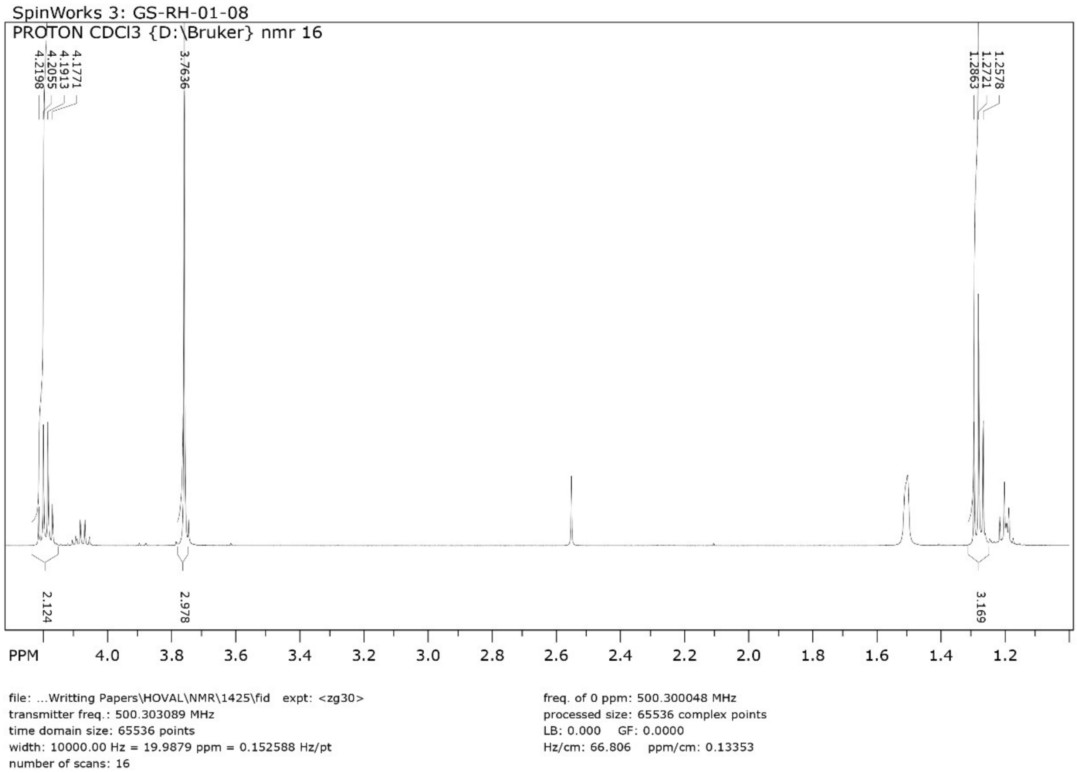

1H-NMR spectrum of Ethyl *(E)*-3-(3-methoxyphenyl)acrylate (Entry 6, Table 6, Expansion 2)

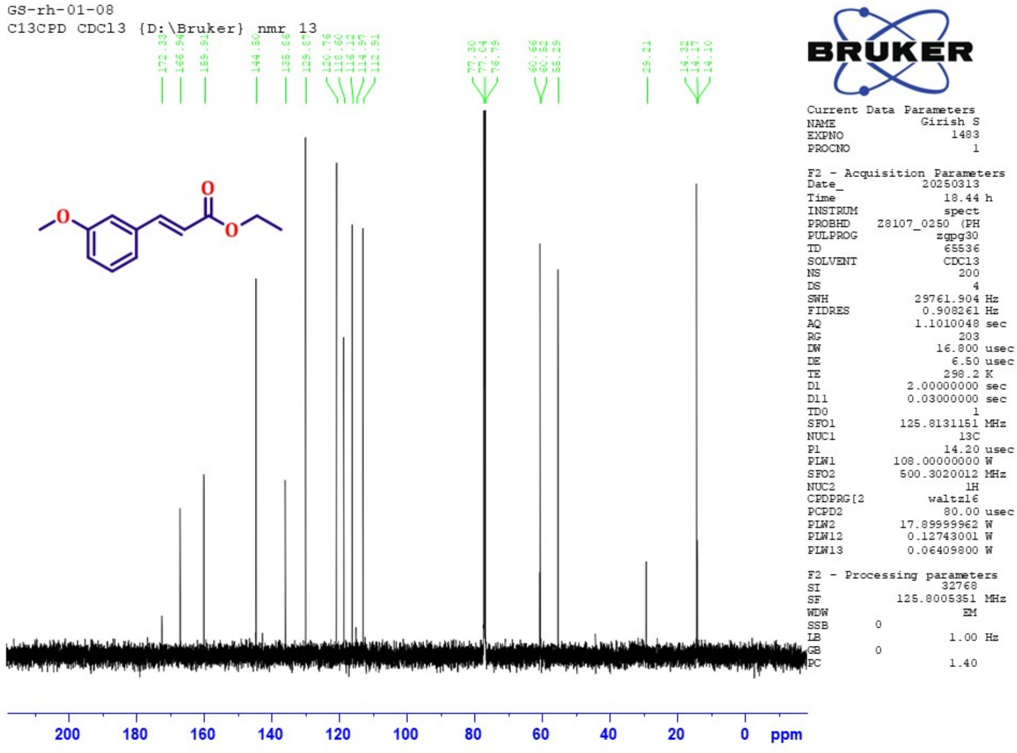

13C NMR spectrum of Ethyl *(E)*-3-(3-methoxyphenyl)acrylate (Entry 6, Table 6)

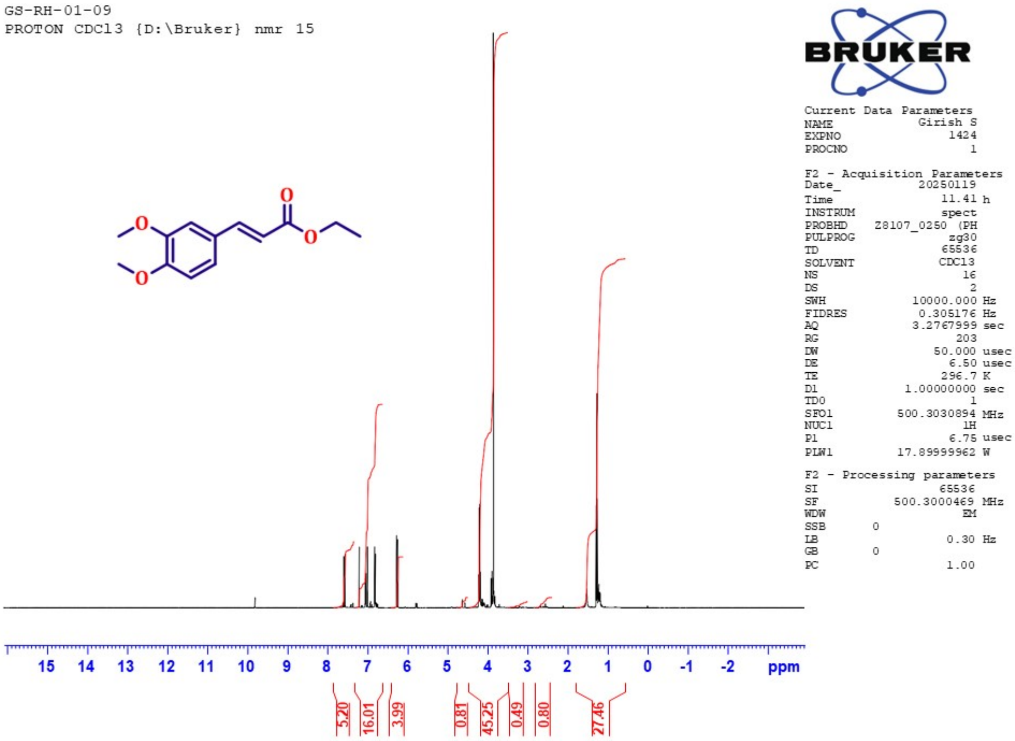

1H-NMR spectrum of Ethyl *(E)*-3-(3,4-dimethoxyphenyl)acrylate (Entry 7, Table 6)

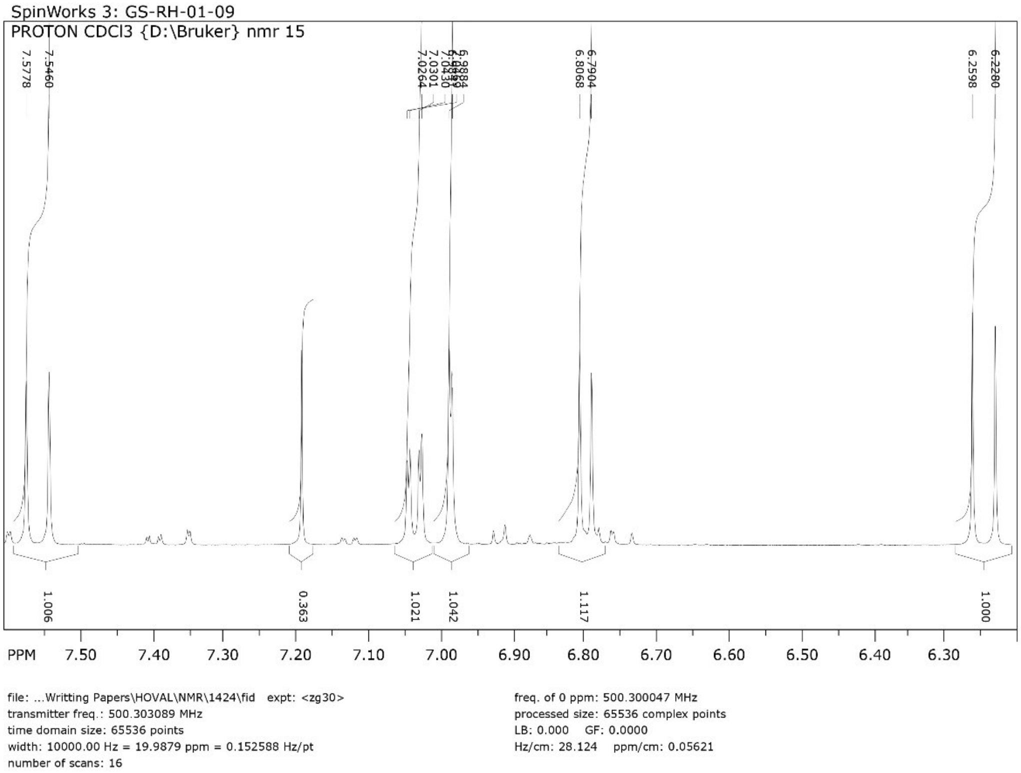

1H-NMR spectrum of Ethyl *(E)*-3-(3,4-dimethoxyphenyl)acrylate (Entry 7, Table 6, Expansion 1)

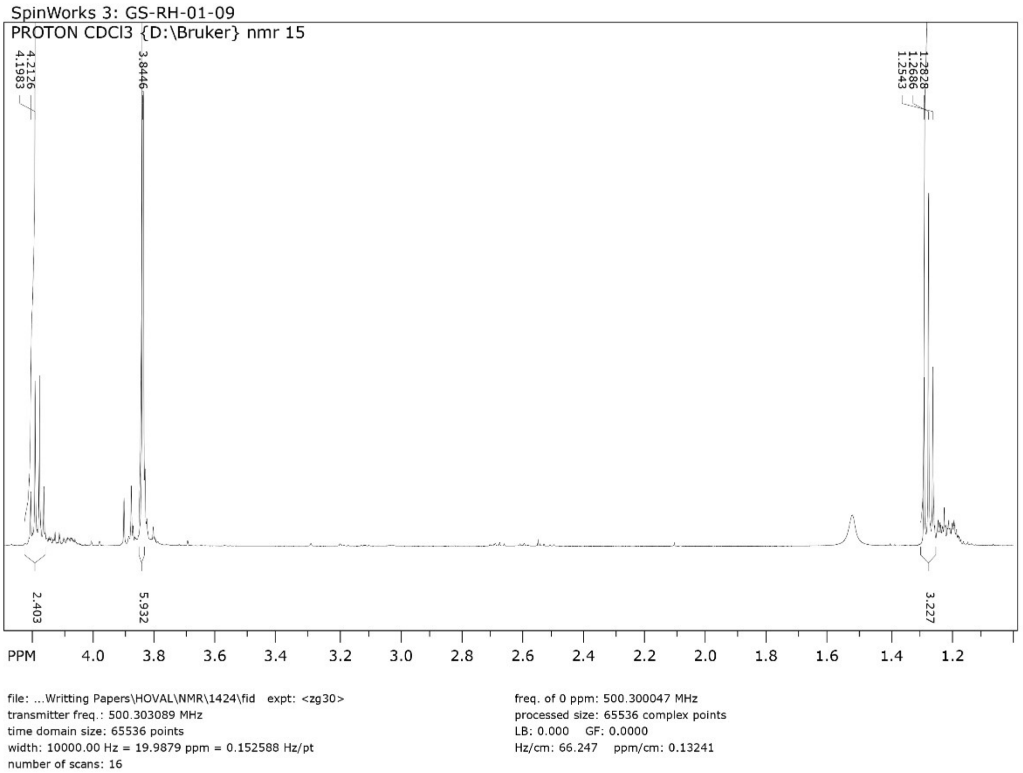

1H-NMR spectrum of Ethyl *(E)*-3-(3,4-dimethoxyphenyl)acrylate (Entry 7, Table 6, Expansion 2)

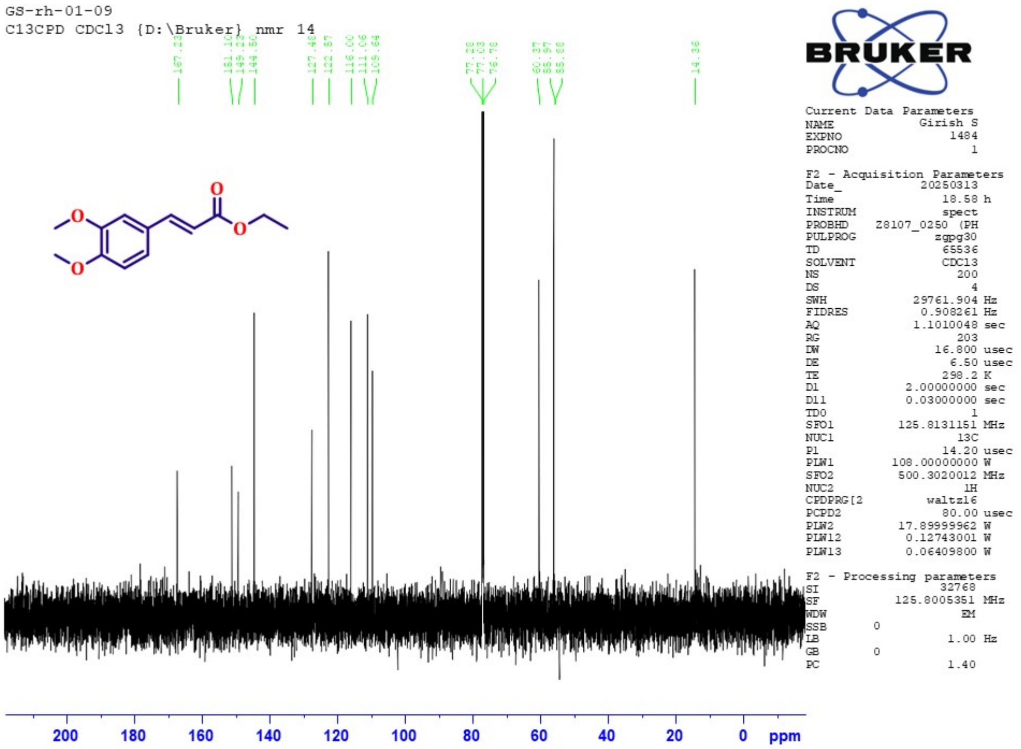

13C NMR spectrum of Ethyl *(E)*-3-(3,4-dimethoxyphenyl)acrylate (Entry 7, Table 6)

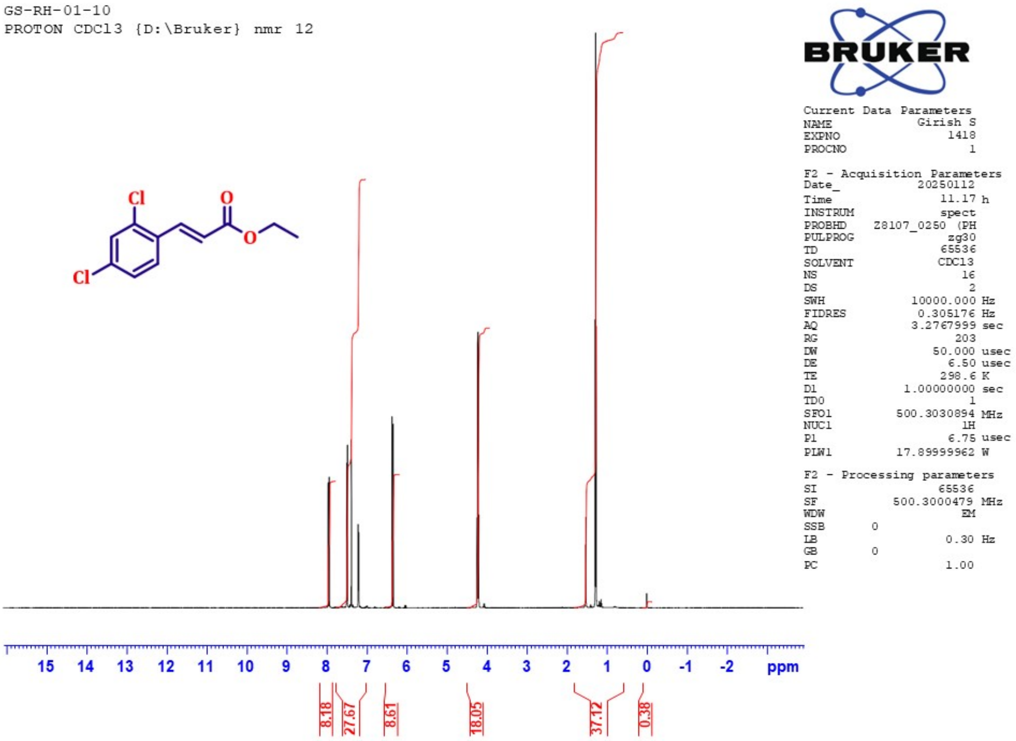

1H-NMR spectrum of Ethyl *(E)*-3-(2,4-dichlorophenyl)acrylate (Entry 8, Table 6)

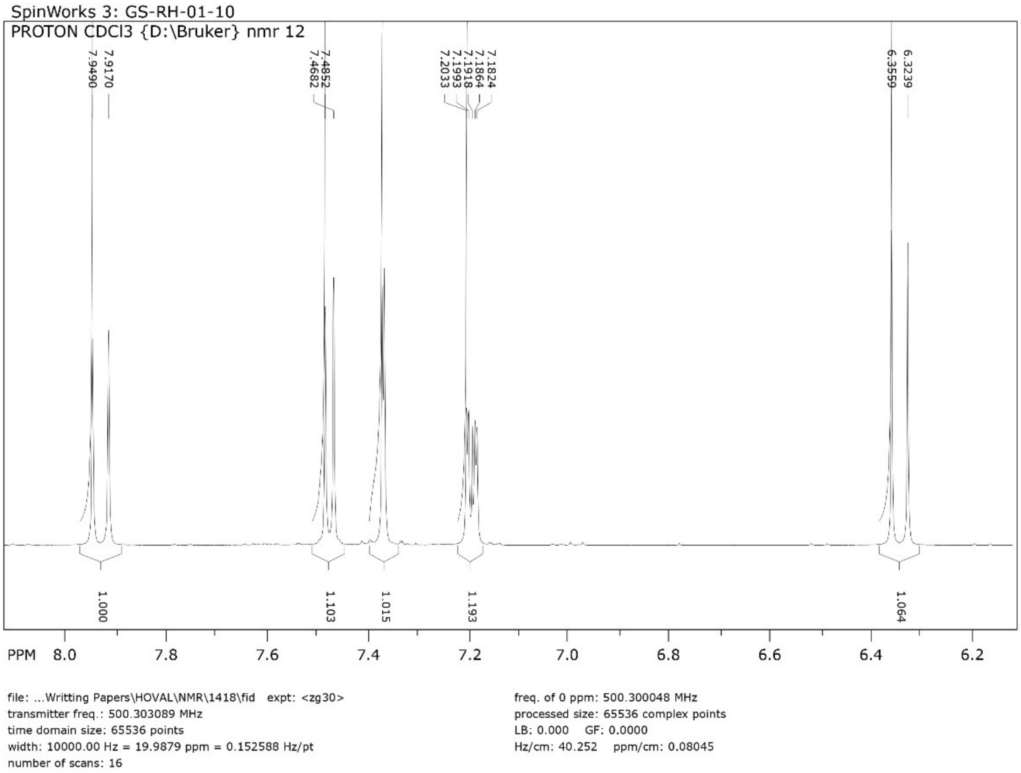

1H-NMR spectrum of Ethyl *(E)*-3-(2,4-dichlorophenyl)acrylate (Entry 8, Table 6, Expansion 1)

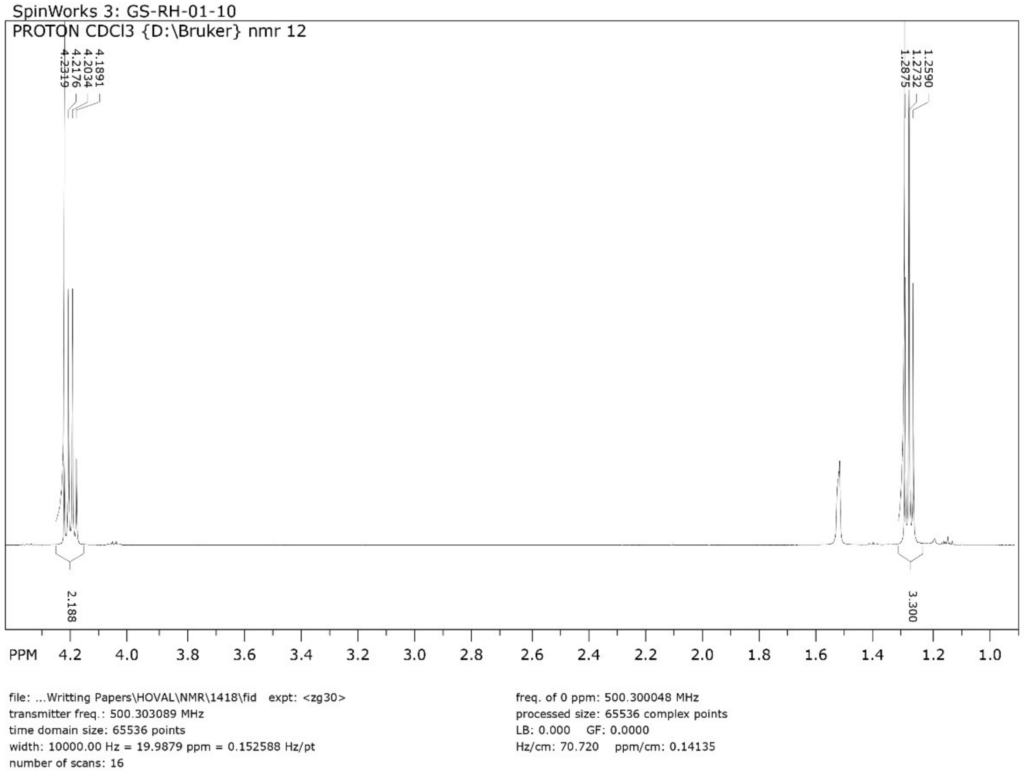

1H-NMR spectrum of Ethyl *(E)*-3-(2,4-dichlorophenyl)acrylate (Entry 8, Table 6, Expansion 2)

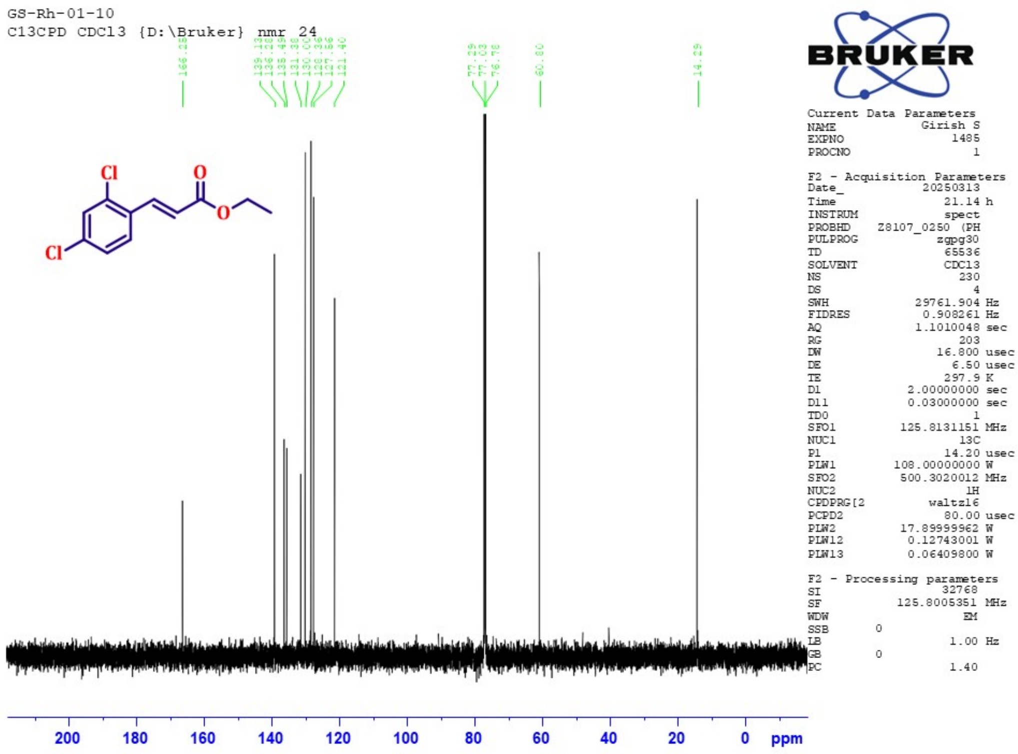

13C NMR spectrum of Ethyl *(E)*-3-(2,4-dichlorophenyl)acrylate (Entry 8, Table 6)

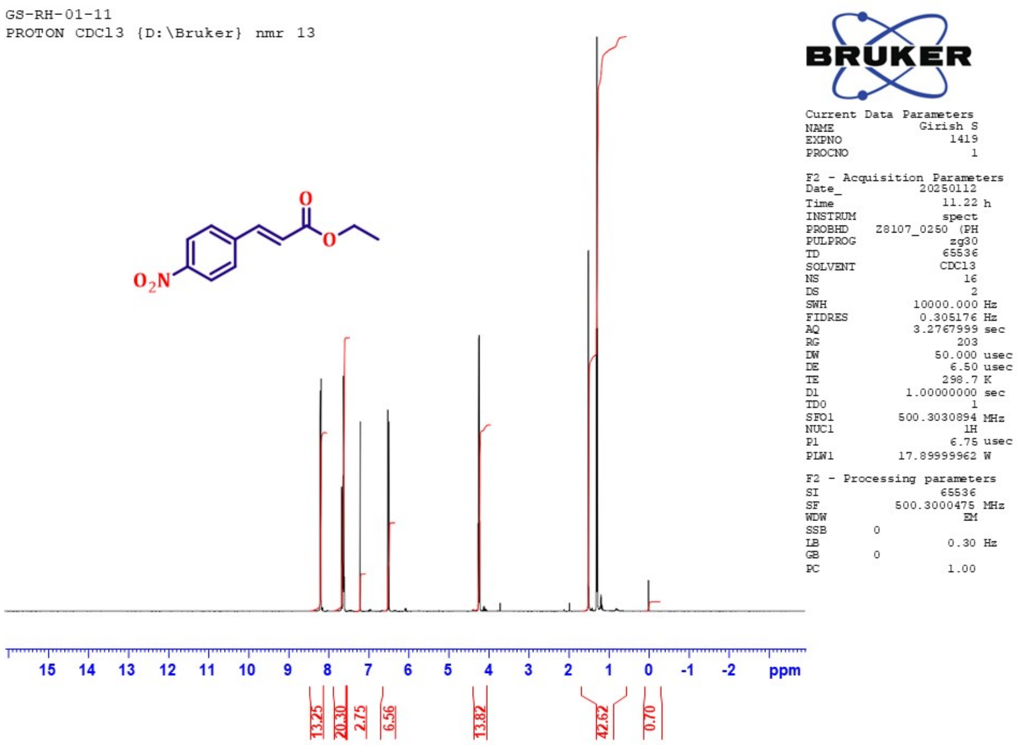

1H-NMR spectrum of Ethyl *(E)*-3-(4-nitrophenyl)acrylate (Entry 9, Table 6)

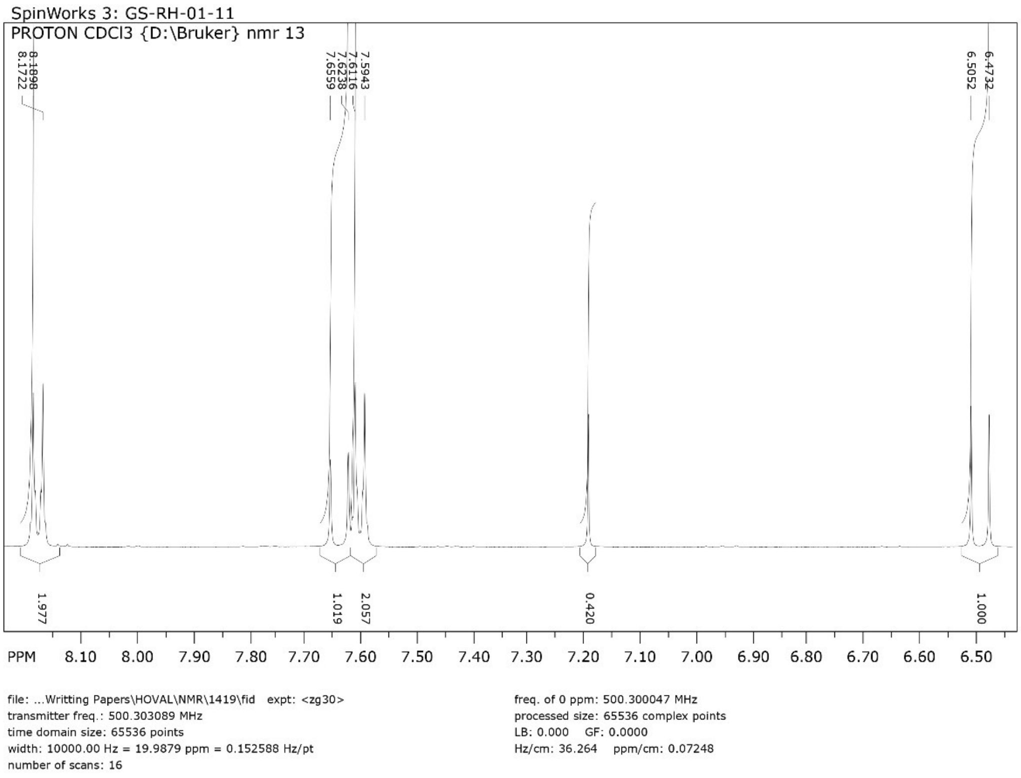

1H-NMR spectrum of Ethyl *(E)*-3-(4-nitrophenyl)acrylate (Entry 9, Table 6, Expansion 1)

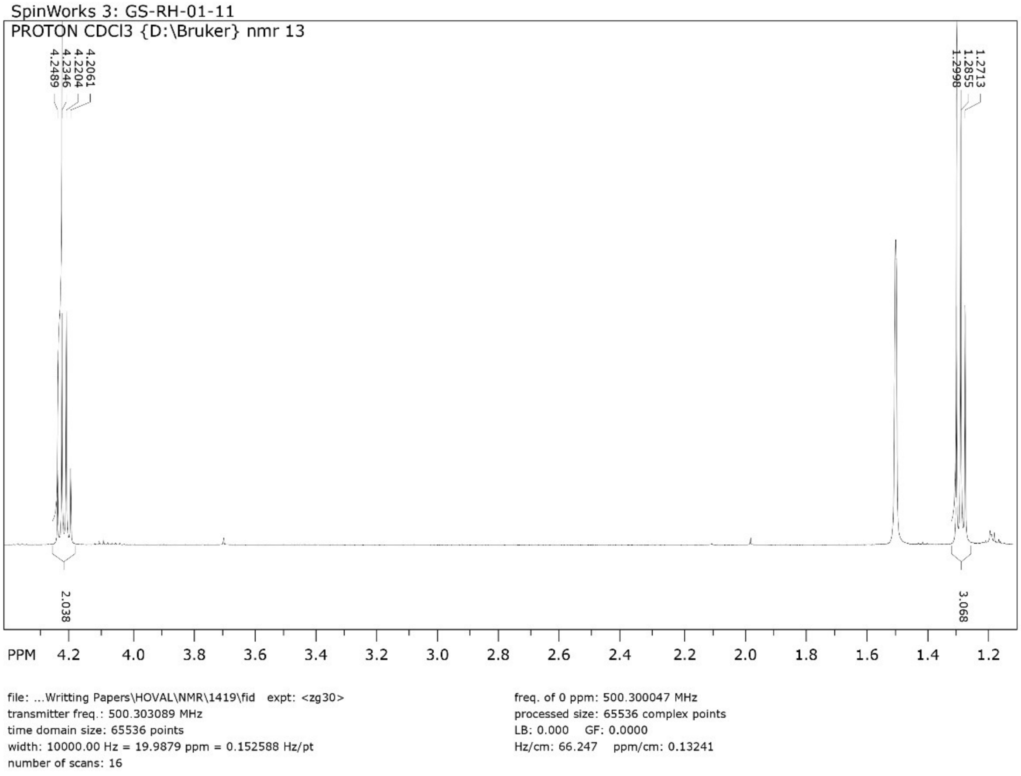

1H-NMR spectrum of Ethyl *(E)*-3-(4-nitrophenyl)acrylate (Entry 9, Table 6, Expansion 2)

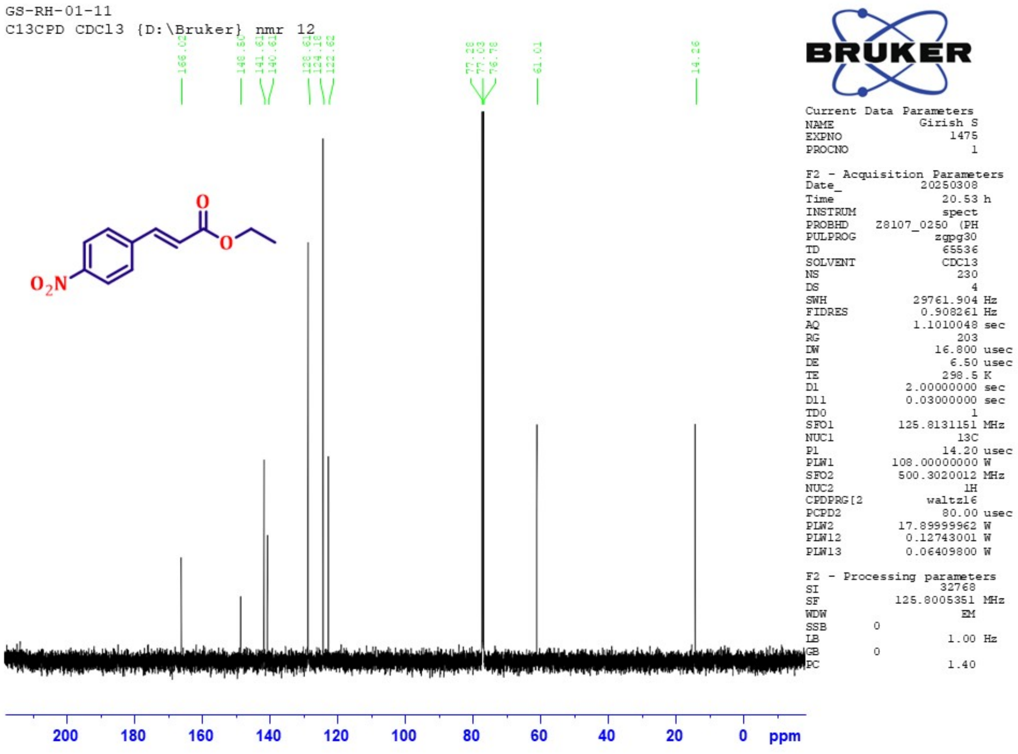

13C NMR spectrum of Ethyl *(E)*-3-(4-nitrophenyl)acrylate (Entry 9, Table 6)

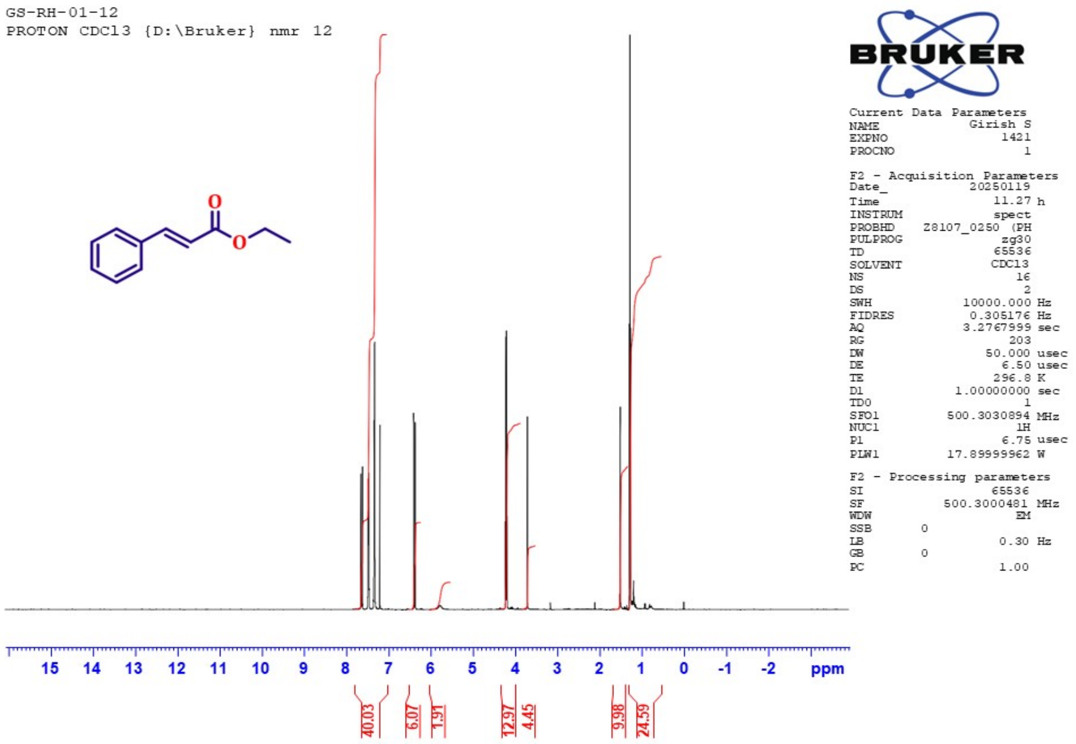

1H-NMR spectrum of Ethyl *(E)*-cinnamate (Entry 10, Table 6)

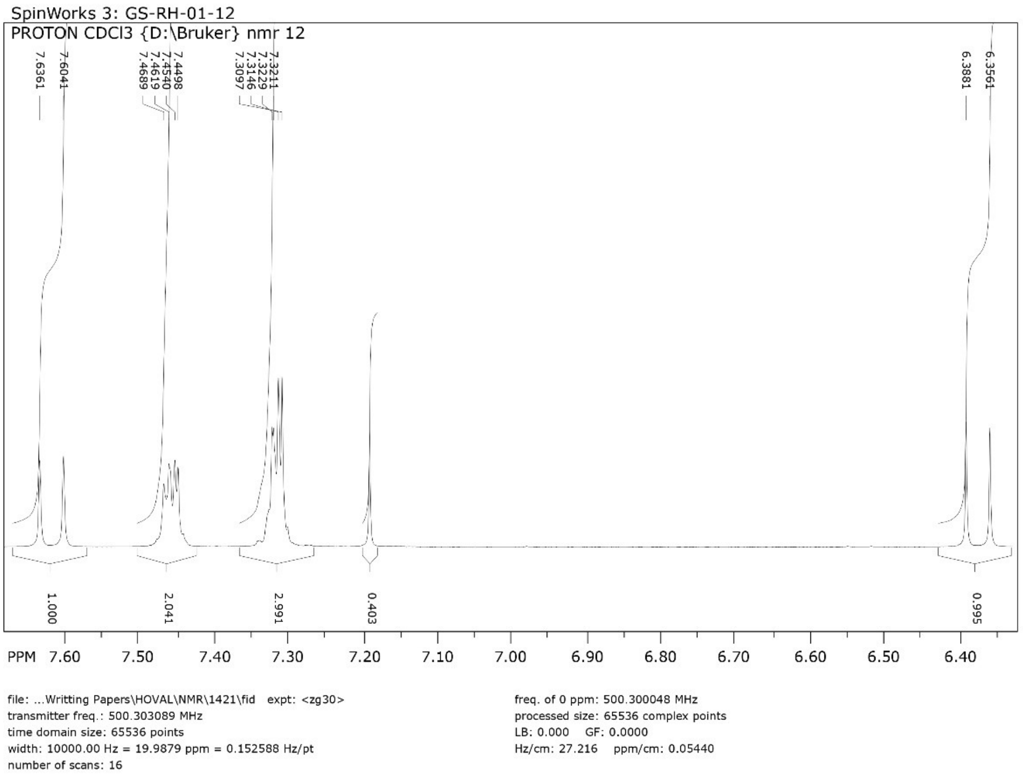

1H-NMR spectrum of Ethyl *(E)*-cinnamate (Entry 10, Table 6, Expansion 1)

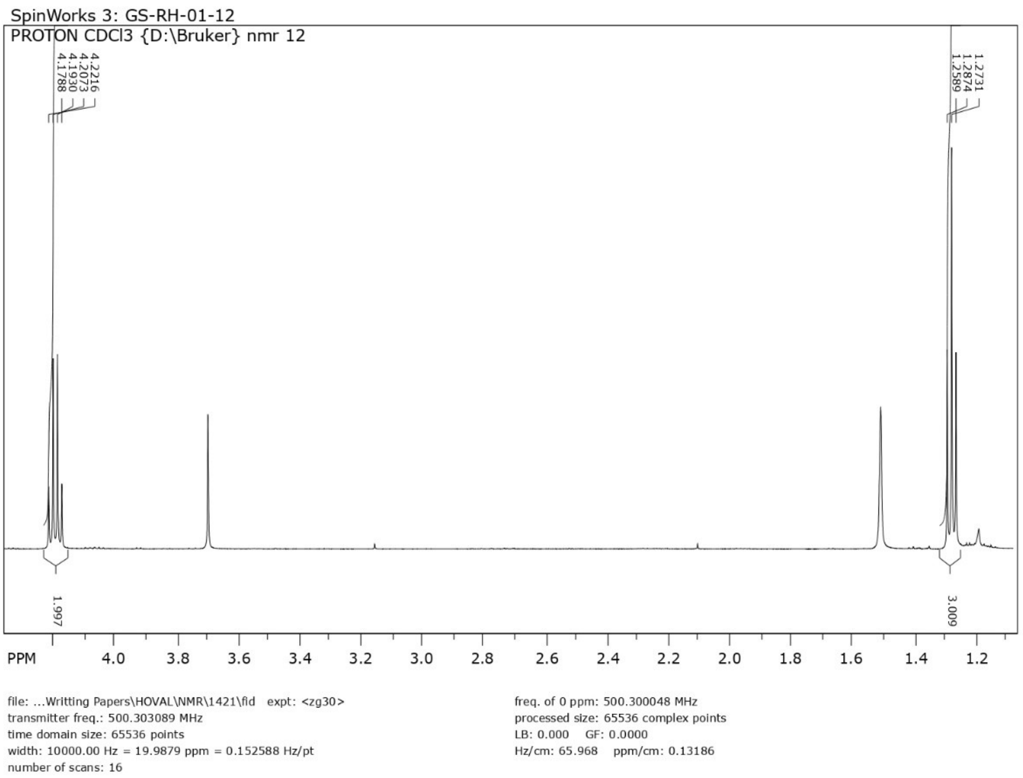

1H-NMR spectrum of Ethyl *(E)*-cinnamate (Entry 10, Table 6, Expansion 2)

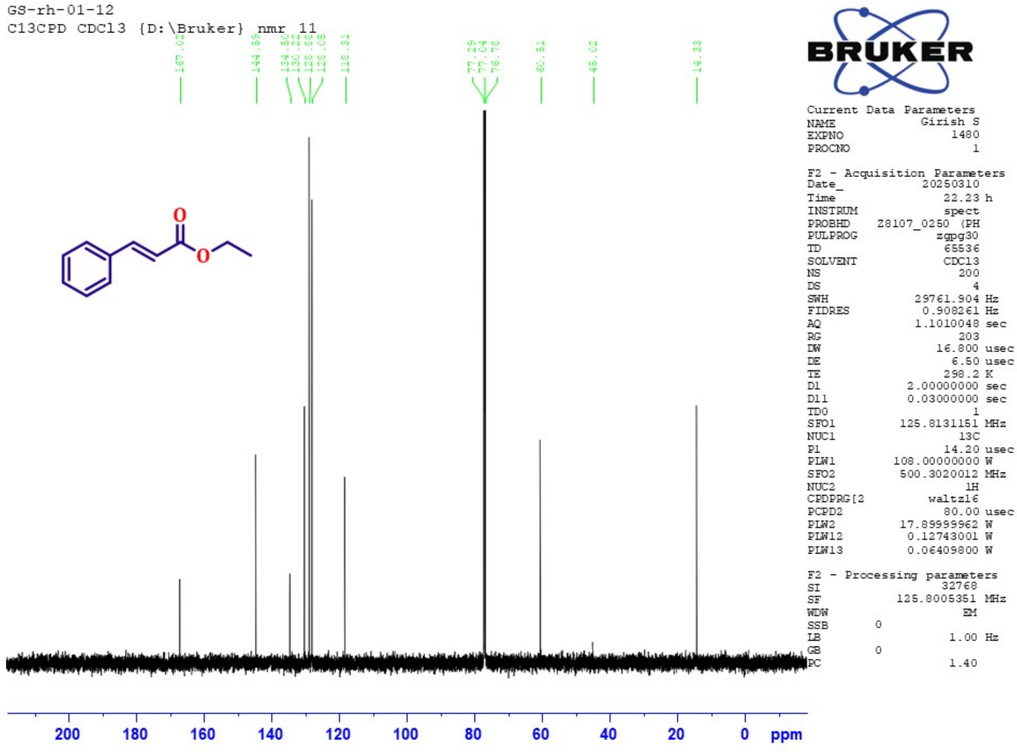

13C NMR spectrum of Ethyl *(E)*-cinnamate (Entry 10, Table 6)

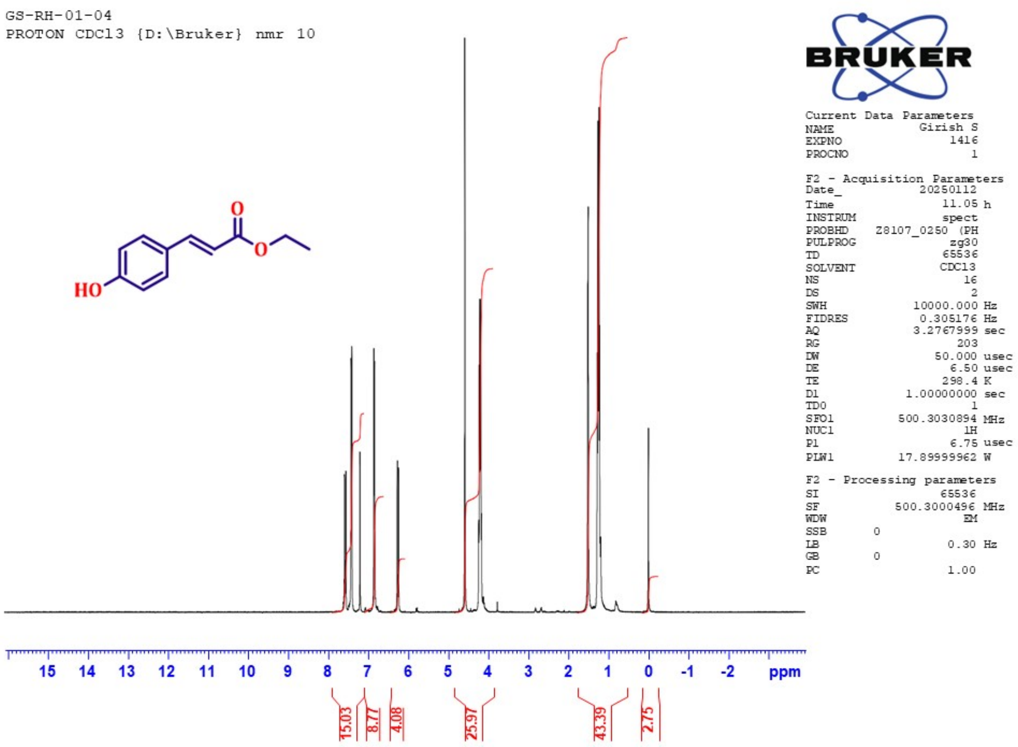

1H-NMR spectrum of Ethyl *(E)*-3-(4-hydroxyphenyl)acrylate (Entry 11, Table 6)

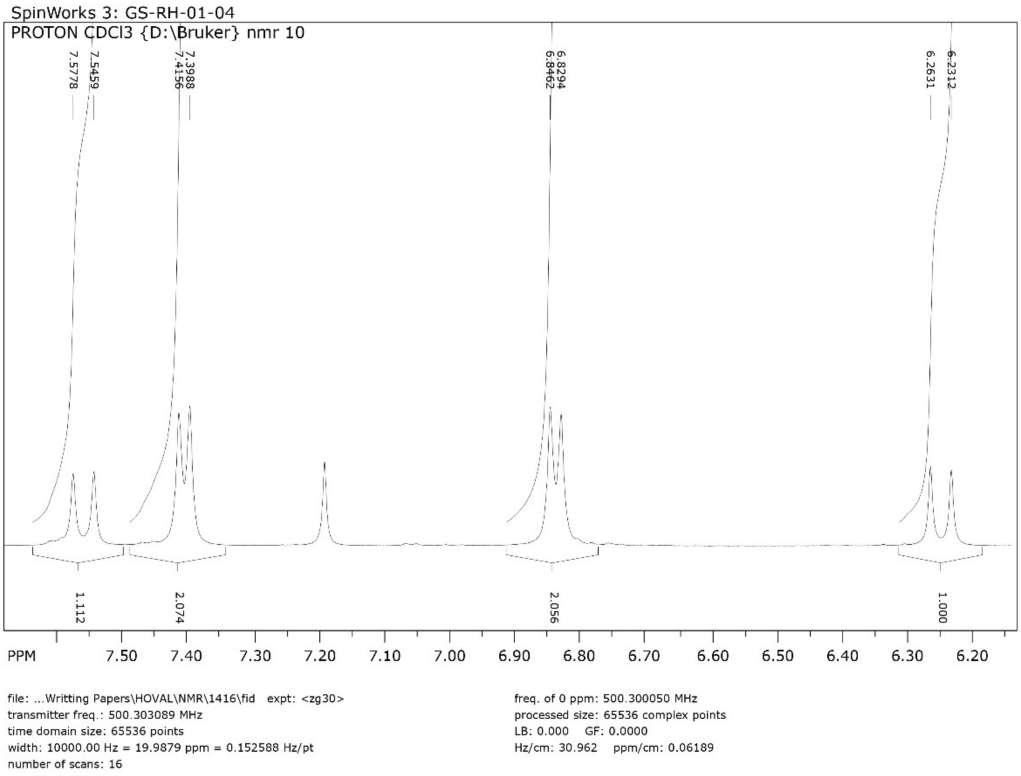

1H-NMR spectrum of Ethyl *(E)*-3-(4-hydroxyphenyl)acrylate (Entry 11, Table 6, Expansion 1)

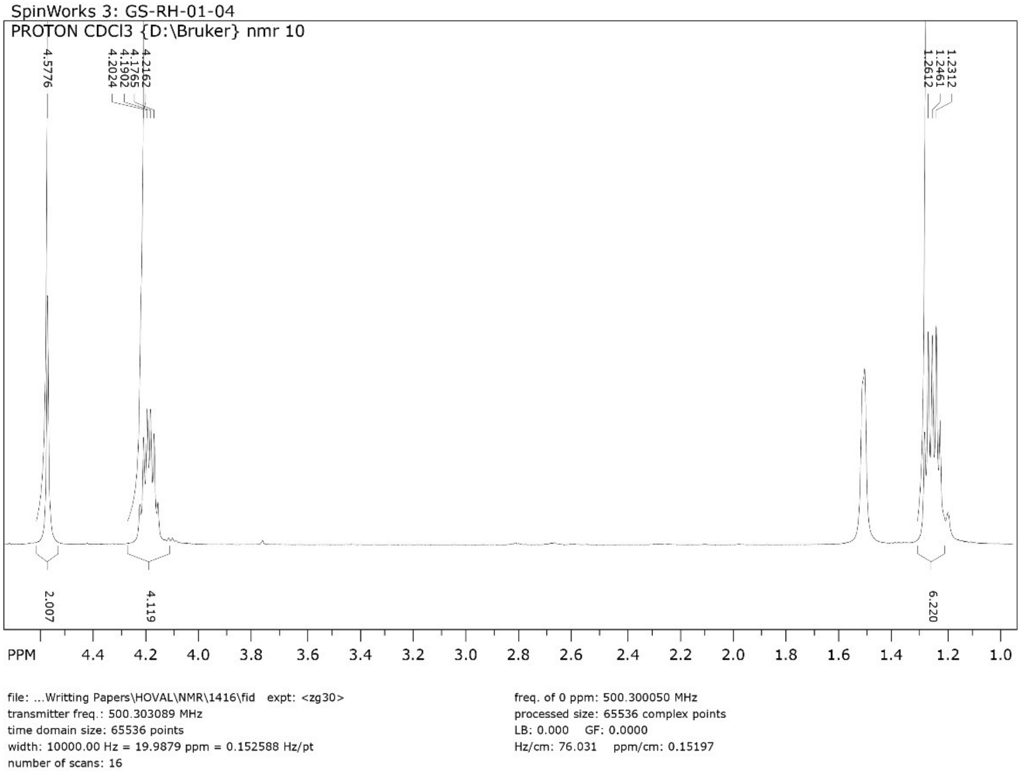

1H-NMR spectrum of Ethyl *(E)*-3-(4-hydroxyphenyl)acrylate (Entry 11, Table 6, Expansion 2)

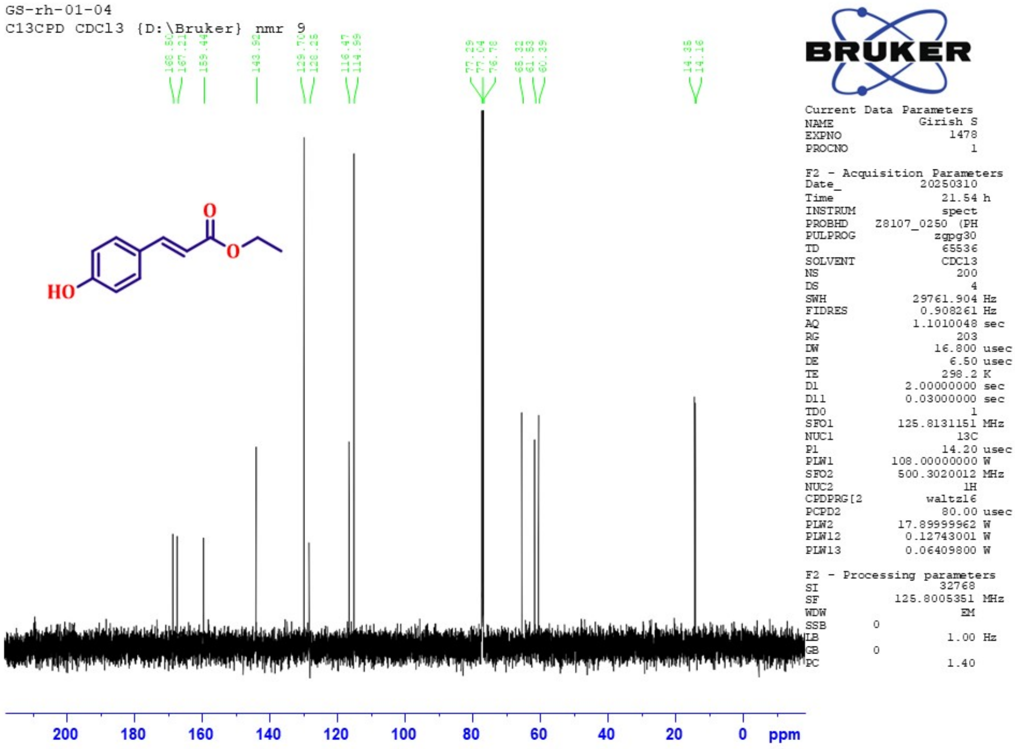

13C NMR spectrum of Ethyl *(E)*-3-(4-hydroxyphenyl)acrylate (Entry 11, Table 6)
